# Supplementary material for: Accelerating protein design by scaling experimental characterization
Source: Nat Commun. 2026 Aug 20;17:9945. doi: 10.1038/s41467-026-76740-9 (PMC13586242; doi:10.1038/s41467-026-76740-9)
Supplement: Supplementary file 1 [file 41467_2026_76740_MOESM1_ESM.pdf]

Supplementary Materials for

Accelerating protein design by scaling experimental characterization

Jason Qian, Lukas F. Milles, Basile I. M. Wicky, Robert J. Ragotte, Amir Motmaen, Andrew J. Borst, Rebecca Skotheim, Sebastian Ols, Brian Coventry, Xinting Li, Ryan D. Kibler, Inna Goreshnik, Marc Expòsit, Karin Loré, Lance Stewart, David Baker

The PDF file includes:
Supplementary Methods
Figs. S1 to S7
Tables S1 to S9

Supplementary Methods

Step-by-step protocol: SAPP

Components and reagents shown underlined refer to specific items listed with their associated catalogue numbers in Supplementary Table 6. Unless stated otherwise, chemicals were sourced from Millipore-Sigma or Fisher Scientific.

Gene fragment sequence design and ordering

Candidate protein sequences are reverse translated and codon optimized with a custom python script that uses the DNA chisel library to optimize the reverse translated sequence for the target organism (e.g. e_coli, h_sapiens), and removes alternative start sites where possible. DNA sequences are also checked for synthesizability with the DNA manufacturer (IDT) using their web API, to ensure that they can be ordered. Furthermore, if the GOI is too long to be ordered as a single eBlock, two compatible DNA fragments are automatically generated by searching the sequence for an optimal GGA overhang from a set of orthogonal overhangs. Finally, cloning adapters for GGA into custom entry vectors (Supplementary Table 1) are added to the sequences. The output provided by the script is a dataframe of all DNA sequences to order, expected sequences on the target plasmid, and an ordering spreadsheet that can be uploaded to the manufacturer (IDT). DNA fragments are ordered resuspended and concentration-normalized in low EDTA TE buffer (10 mM Tris, 0.1 mM EDTA, pH 8.0) in ECHO-qualified 384-well plates.

GGA cloning

Target plasmids must be propagated in a *ccdB* resistant strain, here we use NEB Stable cells. Available GGA vectors are listed in Supplementary Table 1.

Custom GGA vectors
If required, custom GGA entry vectors can be made using template vector LM1369. This general template has a pET backbone, and features PaqCI Type IIS restriction cloning sites for GGA assembly between the start and stop codons, allowing insertion of linear DNA fragments with arbitrary N- and/or C-terminal tags and a *ccdB* cassette to facilitate creation of customized GGA entry vectors for the cloning strategy described above. A custom GGA target vector can thus be assembled in 3 days (Day 1 GGA into LM1369 and plating, Day 2 picking clones for overnight growth, Day 3 DNA prep and sequencing).

Preparing GGA vector stocks
GGA vectors must be propagated in *ccdB* resistant NEB Stable *E. Coli*. In brief, we transform GGA target vector plasmid into NEB Stable according to the manufacturer's protocol, plate on LB-agar plate containing the appropriate antibiotic (e.g. Kanamycin for LM0627) and pick individual clones to inoculate 50 mL of LB media (**not** rich media such as Terrific Broth). Cultures are grown overnight in 250 mL baffled flasks. Cells are harvested by centrifugation,

and DNA is extracted with a midiprep kit, with typical yields in the range of 50 to 100 µg for LM0627, which is sufficient for 2,400 – 4,800 GGA 1µL reactions (2 µg vectors is necessary for one 96-well plate).

For the GGA reaction, a molar ratio of 1:2 (vector:insert) is required.

- For GGA using the ECHO in 1 µL total reaction volume, we provide an interactive spreadsheet calculator that takes into account all parameters to achieve an optimal reaction mixture. The recipe (for NEB enzymes) uses the following ratios:

**Amount**	**Reagent**
0.1 µL	10x T4 Ligase Buffer
1.2 Units	BsaI-HFv2 restriction enzyme
40 Units	T4 DNA Ligase
2-4 fmol	Target GGA vector
4-8 fmol	Linear DNA insert (at 2-fold excess to GGA target vector)
Fill to 1 µL	Nuclease-free H_2O
**Total reaction volume 1 µL**	

- For GGA assembled with multichannel pipettes or individual tubes by hand at 5 µL total reaction volume we provide another interactive spreadsheet calculator. The recipe (for NEB enzymes) uses the following ratios:

**Amount**	**Reagent**
0.5 µL	10x T4 Ligase Buffer
3 Units	BsaI-HFv2 restriction enzyme
100 Units	T4 DNA Ligase
20-30 fmol	Target GGA vector
40-60 fmol	Linear DNA insert (at twofold excess to GGA target vector)
Fill to 5 µL	Cloning-grade H_2O
**Total reaction volume 5 µL**	

Reactions are assembled into 96-well PCR Plates, and spun down briefly (1,000 x g, 30 seconds) to collect reaction mixture at the bottom of each well, covered with aluminum or

transparent foil, and then incubated at 37 °C for 15 minutes, followed by 5 minutes at 60 °C to reduce background.

Transformation and expression

NB: All steps are performed under the flame.
NB: Competent cells must stay as cold as possible and be dispensed directly after they are thawed to avoid delays by having your equipment ready before you start pipetting (mise en place).

For transformation into the *E. coli* expression strain BL21(DE3) 1 µL of the GGA reaction mixture is used.

- If using the ECHO, the 96-well PCR Plate with 1 µL of GGA reaction is directly placed on ice.
- If using the 5 µL reaction approach 1 µL of GGA reaction is transferred to the bottom of each well of a fresh 96-well PCR plate.

Ensure that the plate and reaction mixture are ice cold before proceeding (especially considering the previous 60 °C inactivation step). For optimal cooling and ease of handling we recommend to use a compatible aluminum cooling block for the 96-well PCR Plate.
NB: The transformation is in small volumes, and produces a few hundred CFUs when using commercial competent cells. If using competent cells with lower transformation efficiencies (e.g. lab-made), the amount of cells must be increased accordingly.

Transfer 6 µL of competent BL21(DE3) cells into each well of the 96-well PCR plate using a precooled (stored at -20 °C) dispenser tip of 200 µL total volume on an electronic repeater pipette. Typically three 200 µL aliquots of BL21(DE3) are necessary for one 96-well plate.
Pipetting cells requires careful handling as the cell suspension releases poorly from the pipette tip, we recommend to pipette onto the side of each well aligning the pipette almost horizontally and retracting the tip orthogonal to the well so that the drop releases easily.

The 96-well PCR plate is “hand centrifuged” (rapidly moving the plate inside the aluminum block downwards) to make the competent cell drops settle at the bottom of each well mixing with the GGA reaction product. Failure to complete this step will lead to failed transformations.
The competent cells with the GGA mixture are incubated covered either with a plastic lid (e.g. from a pipette tip box) or aluminum foil on ice for 30 minutes.
The cells are then heat shocked by placing the 96-well PCR plate in a dry block heater at 42 °C for 10 seconds, and then back on ice for 2 minutes.
Subsequently using an electronic repeater 100 µL of room temperature SOC media is added to each well, covered with breathable film, and the plate incubated at 37°C shaking at 1,000 rpm on an orbital plate shaker for 1 hour.

In the meantime:
Prepare simplified 1L of autoinduction media for protein expression under flame as:
- TB-2 (Terrific Broth II powder from MP biomedicals) autoclaved approx. 980 mL
- 2 mL of 1 M $MgSO_4$ (sterile)

- 20 mL of 50x 5052 (sterile filtered, not autoclaved)
- antibiotic, if using Kanamycin: final 50 µg/mL, so use 1 mL of 50 mg/mL kanamycin stock for 1L of media (sterile)

Media can be stored at 4-8 °C in the refrigerator for 2-3 weeks.

Under flame, dispense 1 mL per well of autoinduction media into four 96-well round bottom deep well plates for protein expression using a 1 mL multichannel pipette. The number of expression culture plates may be increased to e.g. six if more protein produced is desired.

If glycerol stocks of the cultures are desired, transfer transformations after recovery into a 96-well round bottom deep well plate containing 1 mL LB media per well with appropriate antibiotic (typically 50 µg/mL kanamycin), cover with breathable film, and incubate for 1 h at 37 °C, shaking at 1,000 rpm on an orbital plate shaker. Then dispense 50 µL into each expression plate to be incubated for 20 h as given below. Continue growing the LB media plate overnight (12-16h) at 37 °C at 1,000 rpm on an orbital plate shaker. Prepare glycerol stocks in a 96-well receiver plate as per well: 100 µL of sterile 50% (v/v) Glycerol in H_2O + 100 µL cultures in LB media (swirl pipette tips in wells to mix) and cover with aluminum foil to be stored at -80 °C.

Transfer 25 µL per well of the transformations after recovery into each well of the 96-well round bottom deep well plates for expression with 1 mL of autoinduction media. Cover plates with breathable film.

Incubate for a minimum of 20 hours (do not shorten this step, your cultures may not induce) at 37˚C on an orbital plate shaker.

Harvest bacterial pellets by centrifuging at 4,000 x g for 5 minutes, discard supernatant by confidently inverting the plates and pat them dry on clean paper towels. Cover with aluminium foil and store at -80 °C or directly proceed to purification.

Immobilized Metal Affinity Chromatography (IMAC)

Prepare buffers:

Lysis buffer is mixed from B-PER chemical lysis buffer containing: 0.1 mg/mL final Lysozyme (from 100 mg/mL stock, in sterile filtered Tris 50 mM 100 mM NaCl pH 7.5, 50% (v/v) glycerol, stored aliquoted at -20˚C), Benzonase (stock diluted as 1:10000, e.g. 4 uL in 40 mL Lysis buffer), cheaper alternative: 0.01 mg/mL final DNAseI, 1mM final PMSF (**NB**: PMSF is toxic!) (from PMSF stock of 100 mM in 2-Propanol, sterile filtered). Add PMSF last as it is not stable in aqueous buffer for long and then use the buffer immediately.

Wash Buffer: 20 mM Tris, 300 mM NaCl, 25 mM Imidazole, pH 8 (can be prepared as 10x stock)

Elution Buffer: 20 mM Tris, 300 mM NaCl, 500 mM Imidazole, pH 8.

Calculate 100 µL per 1mL of culture in well to lyse, i.e. approx. 40 mL of Lysis buffer when prepping 4 full 96-well deep well plates with 1 mL of culture per well for 96 candidates tested.

Add 100 µL of lysis buffer to each well and incubate shaking at 1,000 rpm at 37 °C for 15 minutes to resuspend and lyse pellets. Consolidate everyting into one of the four 96-well round bottom plates using a multichannel pipette.

Spin down to separate the insoluble fraction, 4,000 x g for 15 minutes.

In the meantime prepare plates for IMAC:
Dispense 50 µL of Ni-NTA resin into a 96-well fritted plate, by dispensing 100 µL of a 50% (v/v) resuspended slurry of the resin using a large repeater pipette. Mix the slurry often by inverting its container to prevent it from settling.
Equilibrate resin on vacuum manifold by applying 3 rounds of 400 µL of wash buffer per well, make sure to apply gentle vacuum to not fully dry and crack the resin to prevent channeling.

After centrifugation, carefully collect the 400 µL of supernatant by aspiration from the bottom of the well using a 1000 µL multichannel pipette (taking care not to disturb the insoluble pellet), and directly apply that to the Ni-NTA Resin on the fritted plate, let slowly drip with no vacuum applied for approximately 5 minutes.

Perform 4 rounds of washing the resin with 400 µL of wash buffer per well. As before, be careful not to dry or crack the resin with the vacuum.
Before eluting, blot the plate spouts on Kimwipes multiple times to remove excess wash buffer. Stack from bottom to top: a 96-well receiver plate, a 96-well sterile filtration plate, the 96-well fritted plate with the Ni-NTA resin. Add 200 µL of elution buffer to the 96-well fritted plate resin, incubate for 5 minutes.
Spin down at 1,000 x g for 1 minute to clear all elution buffer from the resin, remove the fritted plate. Spin the sterile filtration plate at 2,000 x g for 2 minutes to filter the samples. Check by eye that all have passed through the filters, sometimes longer centrifugation times may be necessary.
The final output is the 96 samples in the elution buffer, sterile filtered into 96-well receiver plates, ready for SEC.

Purifying from from the insoluble fraction:
For cases where the protein is mostly present in the insoluble fraction (inclusion bodies), the following protocol can be used in addition (or instead) to the protocol described above for purification from the soluble fraction. After lysis, centrifugation and removal of the soluble fraction, add Wash Buffer containing 6 M GdmCl to the insoluble pellet (add the same volume as the soluble fraction volume you just removed). Incubate at room temperature in a shaking incubator (1000 rpm) for 30 min, followed by centrifugation (4,000 x g for 15 minutes). Pipette the supernatant (making sure not to disturb the pellet), and apply to Ni-NTA resin aliquoted in a 96-well fritted plate as described above. After letting the supernatant flow through by gravity, refold the protein directly on the column by gradualing diluting the amount of GdmCl. Using vacuum at each step, and making sure to not dry the resin, apply Wash Buffer containing progressively diluted GdmCl in steps of 400 uL; 3M, 1.5 M , 0.75 M, 0.375 M, 0.18M. Finalize

refolding by washing the resin with a Wash Buffer containing no GdmCl (3 x 400 uL). Proceed to elution as described above.

If His-tag removal is desired:
When using vectors including a SNAC tag ([...]G|SHHW[...], cutsite indicated by "|", e.g. LM627) the His tag can be cleaved and remain on the Ni-NTA resin, so that the eluate will only contain the protein of interest without tags. The protocol is modified as follows: After the Wash steps, add 2 more Wash steps (400 µL per well) using SNAC cleavage buffer. Blot the spouts of the fritted plate using Kimwipes. Seal the bottom of the fritted plate using parafilm: Place a fresh sheet of parafilm over a 96-well deep well plate, then push the fritted plates spout first onto the flat parafilm covering said plate to align the spouts and achieve a watertight seal. SNAC buffer with 2 mM $NiCl_2$ is added to initiate the cleaving on resin. Thoroughly dry and seal top of the wells of the fritted plate using aluminum foil, and incubate shaking overnight. After at least 16 hours of incubation at RT or 37 °C for improved efficiency, collect the flowthrough, as in the protocol above. Continue to SEC.

Ni-NTA resin can be recovered from the fritted plate by adding water to the well with a squirt bottle and rapidly inverting the plate over a collection box, e.g. an empty pipette tip box, multiple times. The collected resin slurry is then collected, concentrated and stored in a large column with a 25 µm frit (Econo column, Biorad) to be stored in 20% (v/v) EtOH until regeneration (see below).

Bulk Ni-NTA resin regeneration can be done using a vacuum line with collection bottle:
NB: Ni ions are a health and environmental hazard, all flowthrough using Ni buffer must be collected in separate containers and disposed of according to local regulations.

- Strip and clean resin with 5 resin bed Ni-NTA Strip Buffer
- Incubate in Strip buffer for 30 min.
- Wash away strip buffer with ultrapure H2O until pH of flowthrough is near neutral (check with pH test strips)
- Do three washes of 3x resin bed volumes of 100 mM $NiSO_4$, let flow through and collect for separate disposal.
- Incubate in $NiSO_4$
- Wash out remaining $NiSO_4$ with ultrapure water, until water runs clear, collect flowthrough for separate disposal
- Store Regenerated resin in 20% (v/v) EtOH at 4 °C

Size-exclusion chromatography

Size exclusion chromatography is performed with 3 mL resin bed volumes columns, Cytiva Superdex 5-150 GL either S75 (3-70kDa) or S200 (10-600 kDa) depending on expected candidate protein size. To process the number of samples presented here (routinely 192 overnight, often only limited by the fraction collector capacity) an autosampler that can automatically draw and inject hundreds of samples onto the chromatography systems is required. We present methods for two instruments that can perform this: A Cytiva Akta pure with

autosampler, and an Agilent 1260 bioinert with multisampler. In both setups injections are pipelined so that the imidazole elution peak elutes in the next injection before the void volume is reached. Fractionation volumes are set to 200 µL. Before each run, columns are calibrated using chromatography standards (for S75 LMW kit, Cytiva ; for S200 HMW kit, Cytiva).

Agilent 1260 Infinity II

The multisampler module on the Agilent 1260 and 1290 FPLC series allows the injection of the samples automatically overnight, cooled to 4 °C. The loop volume for injection should be at least 100 µL.

Samples are injected sequentially at flow rates of 0.65 mL / minute. Maintaining pump pressures that are compatible with the S200 5-150 and S75 5-150 Superdex models with an approximate pressure drop of 40 bars over the column, respecting their maximum pressure limit of 100 bars. Maximum pressure limits are determined for each column according to the manufacturer's specifications.

The fraction collector is filled with 384-well plates (Greiner Masterblock 250 µL capacity).

Akta pure

The Akta pure system will require an autosampler module to inject the samples automatically. To achieve low dead volume when injecting from the autosampler, a re-wiring of the Akta flow path is required. The samples are directly injected from the autosampler onto the SEC column, which then bypasses the column valve and column pressure sensors and connects directly to the UV detector. To minimize the dead volume between the UV detector and the fractionation arm, the tubing of the fractionator is switched to "blue" i.d. 0.25 mm. This "hotwiring" strategy bypasses the column pressure sensors, instead the pump system pressure is used to calculate pressure limits.

Analysis

The integrated data-analysis pipeline starts by generating plasmid maps from the ordered DNA fragment sequences and specified entry vectors. The open reading frame (ORF) containing the gene of interest (GOI) is automatically identified, and used to generate the expression sequence (designed sequence plus vector-specific tags), and calculate protein parameters (molecular weight, molar extinction coefficient, isoelectric point, charge etc…). Batch-exported chromatograms from either the Agilent or Akta system are analyzed using the python packages NumPy (*76*), SciPy (*77*), and Pandas (*78*, *79*), to detect peaks in the protein absorbance signal at 280 nm and convert their retention volume to approximate molecular weight compared to calibration curve assembled from standards (LMW [S75], or HMW [S200], Cytvia). In addition, by integrating the absorbance signal to determine protein concentration in each fraction, the following information can be extracted for each design: total soluble yield, polydispersity, estimated molecular weight and aggregation state of each peak. The data is exported into a standardized open format dataframe (HDF5 (*80*)). Furthermore, an algorithm for automatically picking fractions for pooling was implemented as follows; the user chooses the number of fractions to pool, and the selection logic, which can be based either on the fraction with the

largest integral, or the fraction containing a peak that is closest to the expected retention volume. The list of fractions to pool is then used to generate the robot script.

Robotic pooling

Wells for pooling are selected using the analysis software described above. Samples are pooled and – if desired – concentration-normalized using an OT-2 pipetting robot. The software outputs an OT-2-compatible script for pooling, and a P300 single-channel pipette is used to extract relevant fractions from collection plates, and consolidate them into a 96-well plate. If the option is selected, the robot can add the appropriate amount of buffer to each consolidated well to dilute proteins to a user-defined concentration.

Auxiliary protocols

Ni-NTA resin regeneration:

Used resin is collected in a large glass column (BioRad Econo Column) for batch regeneration. Regeneration requires usage of $NiSO_4$, which is an environmental poison and skin irritant, be sure to follow proper protection measures, and dispose of all Nickel ion contaminated ions according to local regulations. Stripping Buffer (0.5 M NaOH, 100 mM EDTA).

1. Pour stripping buffer until resin is white and all green Ni is eluted
2. Cap column, incubate resin in stripping buffer as a slurry on rotator for 30 minutes
3. Pour ultrapure H_2O over resin until water on resin till pH is neutral (check with pH test strips)
4. Pour green ($NiSO_4$ 100 mM in H_2O) solution until the entire resin is blue again and no white spots remain. Collect flow-through as nickel Waste (toxic).
5. Rinse with ultrapure H_2O until eluate runs clear, collect eluate as nickel waste (toxic).
6. Adjust resin slurry to 50% resin (v/v) in 20% EtOH (v/v) for use in SAPP protocol.
7. Store regenerated resin 20% EtOH (v/v). Store at 4˚ C.

SEC Column cleaning

After each run SEC columns are cleaned as follows; SEC column cleaning downflow at 0.5 mL / minute if not otherwise declared (CV = Column volume). All buffers are sterile filtered (0.2 µm).
Downflow clean:

- 1 CV ultrapure H2O
- 1 CV 0.5 M NaOH at 0.2 mL / minute
- 1 CV ultrapure H2O
- 1.1 CV 20% (v/v) EtOH in H_2O to store

Regularly, if the pressure drop over the column increases with time, an upflow clean is required to rinse the top column filter.
Upflow clean on Akta using the column loop in upflow, on Agilent HPLC by manually inverting column direction:

- Upflow: 1 CV ultrapure H2O 0.25 mL / minute
- Upflow: 1 CV 0.5 M NaOH at 0.13 mL / minute
- Upflow: 1 CV ultrapure H2O 0.25 mL / minute
- 1.1 CV 20% (v/v) EtOH in H2O for storage 0.25 mL / minute

Step-by-step protocol: DMX

Components and reagents shown underlined refer to specific items listed with their associated catalogue numbers in Supplementary Table 8. Unless stated otherwise, chemicals were sourced from Millipore-Sigma or Fisher Scientific.

UMI and primers sequences can be found in Supplementary Table 3 and 4.

Schematic of library entry into the multi-purpose DMX vector is shown in Supplementary Fig. 4.

Day 1: Library amplification from oligo pool, and cloning into DMX vector

1.1) Library amplification from oligo pool

The library is amplified from an oligo pool by PCR (25 µL) with corresponding forward and reverse primers (containing BsmBI cut sites: CGTCTCgagga at 5' end and ttccgGAGACG at 3' end) (Supplementary Table 4) for 14 cycles.

**Amount**	**Reagent**
12.5 µL	2x KAPA HiFi HotStart Ready Mix
1.25 µL	20x EvaGreen
0.75 µL	Forward library primer (10 uM)
0.75 µL	Reverse library primer (10 uM)
2.5 ng	Oligo library DNA
Fill to 25 µL	Nuclease-free H_2O

**PCR program**	**Time**
95°C	3 minutes
98°C	20 sec | 14 cycles
65°C	15 sec |
72°C	40 sec |
72°C	1 minute

4°C	Hold

The amplified library is run on a 2% agarose gel, and the corresponding band extracted with Zymoclean Gel DNA Recovery Kits (D4007/D4008, Zymo Research), eluted into 8 µL of elution buffer; 1 µL of the eluant was quantified using Qubit (Invitrogen) with dsDNA Quantitation, High Sensitivity (Q32851, Invitrogen). Expect the yield after gel extraction to be ~100 - 200 ng per PCR reaction.

1.2) Cloning into DMX vector

The amplified library is cloned into the DMX vector by GGA (40 uL reaction). A molar ratio of 3:1 (library:DMX vector) is used to calculate the amount of amplified library to add into the GGA reaction.

**Amount**	**Reagent**
40 Units	BsmBI-v2 NEBridge Golden Gate Assembly kit
4 µL	10x T4 DNA ligase buffer
0.1 pmol total	DMX vector
0.3 pmol total	Purified oligo library DNA
Fill to 40 µL	Nuclease-free H2O

**GGA program**	**Time**
42°C	60 minutes
60°C	5 minutes
4°C	Hold

The GGA product is purified using DNA Clean & Concentrator-5 (D4003, Zymo Research), eluted with 10 µL of nuclease-free H2O, then quantified using a Qubit (Invitrogen) with dsDNA Quantitation, High Sensitivity.

The GGA cloned library (90 ng) is electroporated into 50 µL of *E. Cloni* EXPRESS BL21(DE3) electrocompetent cells (Lucigen) according to the manufacturer's protocol. The cells are recovered in 1 mL in Recovery Medium at 37 °C for 1 hour in a shaking incubator at 250 rpm. **NB:** Expect the time constant from the electroporation to be ~ 4.1 ms.

Recovered cells were serially diluted (1:12,500, 1:25,000, 1:50,000) and plated on round 10-cm LB-Agar dishes containing 100 µg/mL of carbenicillin and grown overnight at 37 °C; the rest of the non-diluted recovered cells were stored at 4 °C overnight for platting the next day.

Day 2: CFU counting and colony generation

The number of colonies are counted from each dilution plate, and multiplied by the dilution factor to calculate the CFUs in the original library. The electroporation efficiency (CFU x library complexity) should >300 to ensure adequate coverage.

The optimal colony picking density for a 25-cm Square BioAssay agar plate (431111, Corning) is ~2,500 colonies. The volume needed from the recovered cells, previously stored 4 °C, is calculated based on the CFU, then were mixed with SOC medium to a final volume of 500 µL, which is plated using beads onto a BioAssay plate containing 100 µg/ml of carbenicillin, and grown overnight at 37 °C. **NB:** For home-made BioAssay plates, it is important to dry the agar plates sufficiently (~30 minutes) to avoid smearing of grown colonies.

Day 3: Colony picking

Colonies are picked using a QPix XE Microbial Colony Picker (Molecular Devices) into ECHO-qualified 384-well plates (#c74290, Beckman Coulter) containing 60 µL of low-salt LB liquid medium and 100 µg/ml of carbenicillin per well.

**Amount**	**Low-salt LB media**
10 g	Tryptone
0.5 g	NaCl
5 g	Yeast Extract
Fill to 1 L	H2O

After picking, the plates are sealed with Breathe Easier plate seals (NC1664397, Fisher Scientific), and grown at 37 °C overnight in a shaking incubator at 1,000 rpm.

If a colony picker is not available, colonies can be manually picked into ECHO-qualified 384-well plates for demultiplexing smaller libraries (<500 genes).

Day 4: GGA barcoding and Nanopore sequencing

4.1) GGA barcoding in cell-lysate

Cells (2 µL) from confluent culture plates are transferred and compressed from four ECHO-qualified 384-well plates and into one polypropylene 1536-well plate (782270, Greiner) plate using an ECHO 525 acoustic liquid handling robot (Beckman Coulter). **NB:** prior to transfer with ECHO, it is important to invert the 384-well cultures plates for 30 minutes to allow cells to gather at the meniscus to maximize cell transfer. After the ECHO transfer, the 1536-well plates are sealed with Axygen® Foil Plate Seal (PCR-AS-600, Fisher) and inserted into a vacuum sealed bag then heat-lysed in a 98 °C water bath for 30 minutes.

A custom python script was used to: 1) randomly generate the four position-specific DNA barcode combinations and calculate their transfer volume; 2) generate the H_2O transfer volume (0.5 µL - volume of barcodes) (see Jupyter notebook in DMX github repository). The ECHO is used to transfer the H_2O volume followed by barcode combinations (2 ng of each of the 4 UMI for a total of 8 ng) into each well of the heat-inactivated 1536-well plates.

GGA mastermix volume (0.5 µL / well) is calculated for all the 1536-well plates and transferred using the ECHO into each well.

**Amount**	**GGA mastermix reagents for one well**
0.4 µL	10x T4 Ligase Buffer
1.2 Units	BsaI-HFv2 restriction enzyme
40 Units	Salt-T4 DNA Ligase
Fill to 0.5 µL	Nuclease-free H2O

The total volume in each well is 3.5 µL (i.e. cell lysate, barcodes, GGA mastermix).

**Amount**	**Reagent for one well**
2 µL	Cell-lysate
0.5 µL	Barcode combination (2 ng / UMI)
0.5 µL	Nuclease-free H2O
0.5 µL	GGA mastermix
**Total reaction volume 3.5 µL**	

**GGA program**	**Time**
37°C	60 minutes

60°C	5 minutes
4°C	Hold

After GGA reaction, the 1536-well plates are inverted and centrifuged at 200 x g into a reservoir to pool all GGA products (i.e. 4 position-specific barcodes ligated with the GOI from each well). The pooled GGA products are purified using Zyppy Plasmid Miniprep (D4036, Zymo Research) according to the manufacturer's protocol, eluted with 20 µL of elution buffer, then quantified using a Qubit (Invitrogen). **NB:** Given that the amount of pooled DNA from the plates may saturate a Zyppy column, it is recommended to split the DNA purification across multiple columns (2 columns per 1536-well plates).

Purified DNA is amplified with the forward/reverse primer pairs (DMX1/DMX2, DMX3/DMX4, DMX5/DMX6) (Supplementary Table 4) in separate PCR reactions (25 µL).

**Amount**	**Reagent**
12.5 µL	2x KAPA HiFi HotStart Ready Mix
1.25 µL	Forward primer (10 uM)
1.25 µL	Reverse primer (10 uM)
20 ng	Purified DNA
Fill to 25 µL	Nuclease-free H2O

**PCR program**	**Time**
95°C	3 minutes
98°C	20 sec | 10 cycles
65°C	15 sec |
72°C	40 sec |
72°C	1 minute
4°C	Hold

The amplified bands from the different PCR reactions are purified by gel electrophoresis (1% agarose) and extracted using Zymoclean Gel DNA Recovery Kits (D4007/D4008, Zymo

Research), eluted with 9 μL of elution buffer, pooled together, then quantified using a Qubit (Invitrogen) with dsDNA Quantitation, High Sensitivity (Q32851, Invitrogen).

4.2) Nanopore long-read sequencing

Sequencing libraries were prepared from amplified barcoded pools (200 fmol) using Ligation Sequencing Kit V14 (SQK-LSK114, Oxford Nanopore Technologies) and loaded onto MinION Flow Cell R10.4.1 (FLO-MIN114, Oxford Nanopore Technologies) according to the manufacturer's protocol. **NB:** for smaller libraries, a Flongle (FLO-MIN114/FLO-FLG114, ONT) can be used instead.

Generation of consensus sequences (Supplementary Fig. 6).

Scripts and tools are available on GitHub. An example dataset and a Jupyter Notebook are provided on GitHub allowing users to run the pipeline on their own.

Software version:

- python (3.11.3)
- dorado (0.9.1)
- samtools (1.21) (*81*)
- chopper (0.9.0) (*91*)
- nanoq (0.10.0) (*92*)
- cutadapt (4.9) (*93*)
- minimap2 (2.28) (*94*)

User input file requirements:

- Nanopore sequencing reads (.pod5)
- UMI and barcode combination sequences (.csv)
- Reference library sequences (.fasta)

Steps:

0) Basecalling

- Dorado: Nanopore sequencing raw reads comprise multiple .pod5 files. Super accuracy model (sup) from dorado was used to convert .pod5 files into .bam files.

1) Read Quality filtering

- Samtools: merge all the .bam files into one, then convert the .bam file into .fastq file.
- Chopper: combination of NanoFilt and NanoLyse that is used to filter sequences based on average read quality and length and outputs a .fastq file. We used a >Q15 cutoff and read length of 400 - 1000 bp.

- Nanoq: output summary report for nanopore reads and filtering.

2) Barcode Demultiplexing

- Cutadapt: demultiplex barcoded reads into individual .fastq file. The users supply lists of i) reference UMI sequences (.csv); ii) barcode combinations of 3 or 4 UMI used for each barcoded well (.csv); iii) primer pairs used for PCR amplifying the final pooled barcoded library

  # Structure of post-PCR barcoded amplicons for 4 UMI:
    - primer pair 1/2: 2-3-4-dmx7-design-dmx0-1
    - primer pair 3/4: 3-4-dmx7-design-dmx0-1-2
    - primer pair 5/6: 4-dmx7-design-dmx0-1-2-3

  # Structure of post-PCR barcoded amplicons for 3 UMI:
    - primer pair 1/2: 2-3-dmx7-design-dmx0-1
    - primer pair 3/6: 3-dmx7-design-dmx0-1-2

  Dmx0 and dmx7 are universal primers flanking design sequence.

  Cutadapt algorithm searches and trims UMI from barcoded reads that match reference UMI sequences (e.g. minimum overlap of 20 out of the full length 25 bp of each UMI), then outputs trimmed reads into file (.fastq) with the name of the well from which it originated.

3) Alignment to Reference and Consensus Sequence Generation

- Minimap2: aligns reads from each file to the reference gene library sequences using a modified Smith-Waterman alignment algorithm and outputs a .sam file.
- Samtools: converts .sam file into .bam file and a .bami index file, then generates consensus sequence (.fa) for each well if read depth is >150 and a base is included in the consensus if at least 51% of reads support it.

4) Perfect Design Filtering

- Python script: builds a list of wells with perfect sequence to re-array by filtering out any wells that 1) contain multiple consensus sequences; 2) do not perfectly match to a sequence in the reference library. Finally, if multiple wells have the same sequence, then only keep one of them.

Supplementary Figures

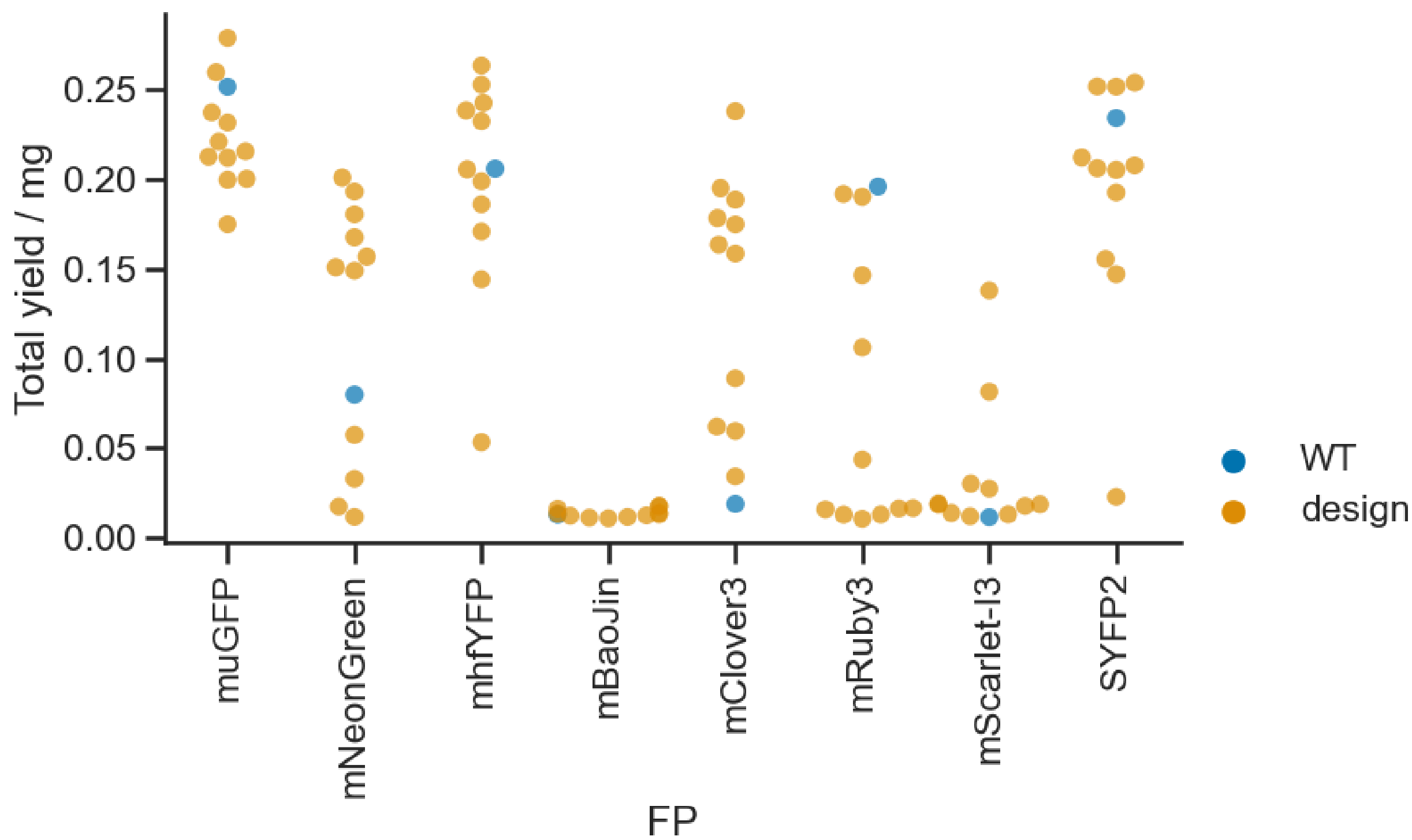

Supplementary Fig. 1. Total soluble yield obtained from SAPP (4 mL input culture) for FP designs, grouped by parental FP (N=11 designs, shown in orange and N=1 WT shown in blue).

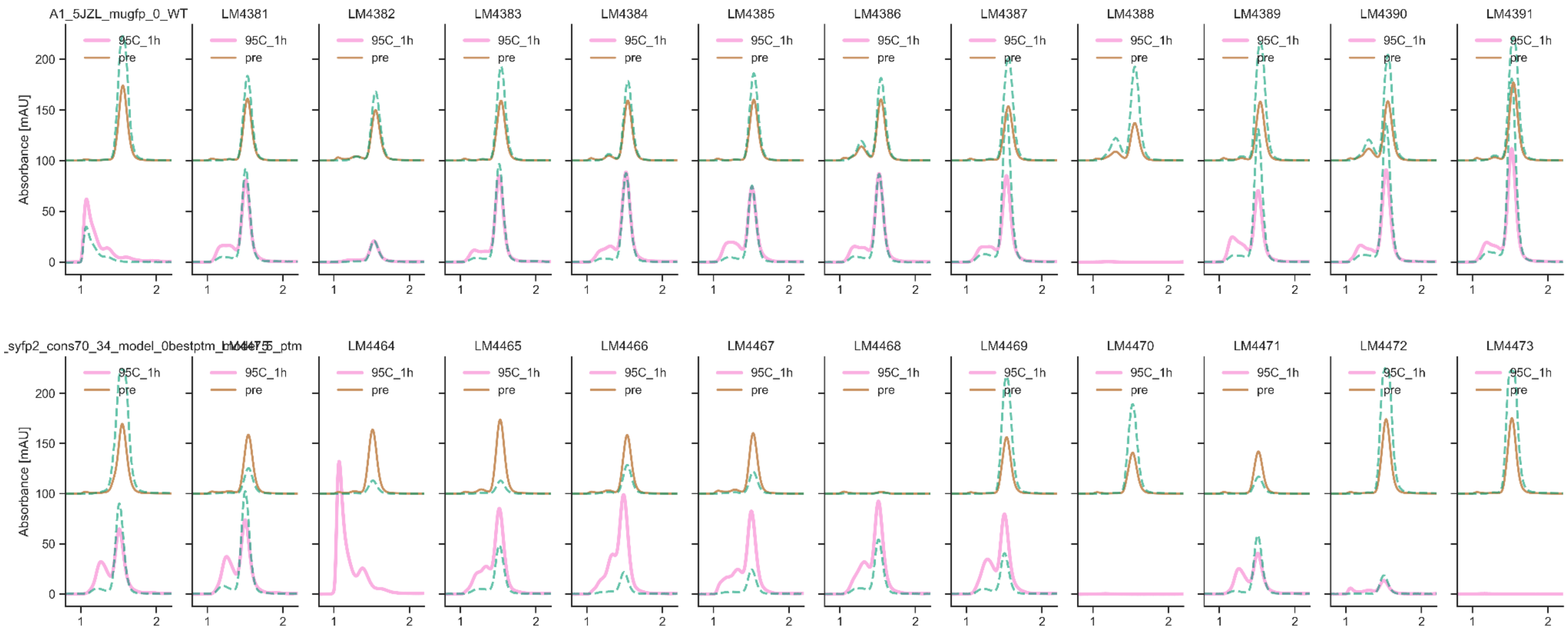

Supplementary Fig. 2. FP SEC chromatograms (x axes in mL) for muGFP redesigns (top) and SYFP2 redesigns (bottom) pre and post 1h incubation at 95 C. Full lines show the absorbance at 280 nm, and dashed lines show the chromophore absorbance (top at 490 nm for muGFP, bottom at 515 nm for SYFP2).

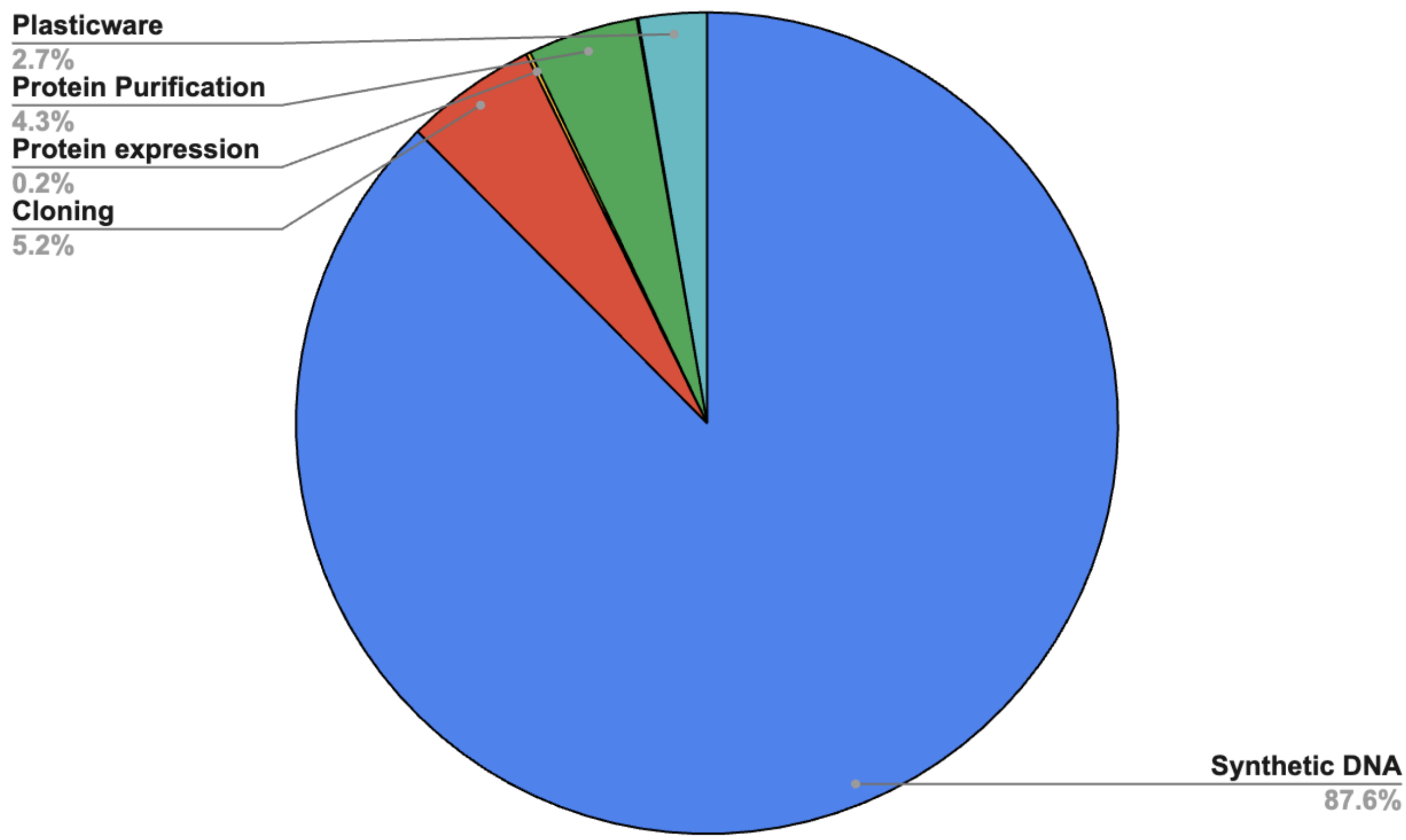

Supplementary Fig. 3. Cost breakdown for end-to-end execution of SAPP protocol starting from a 300 bp gene fragment (cloning/expression/purification/characterization). The total cost for one design is $24.

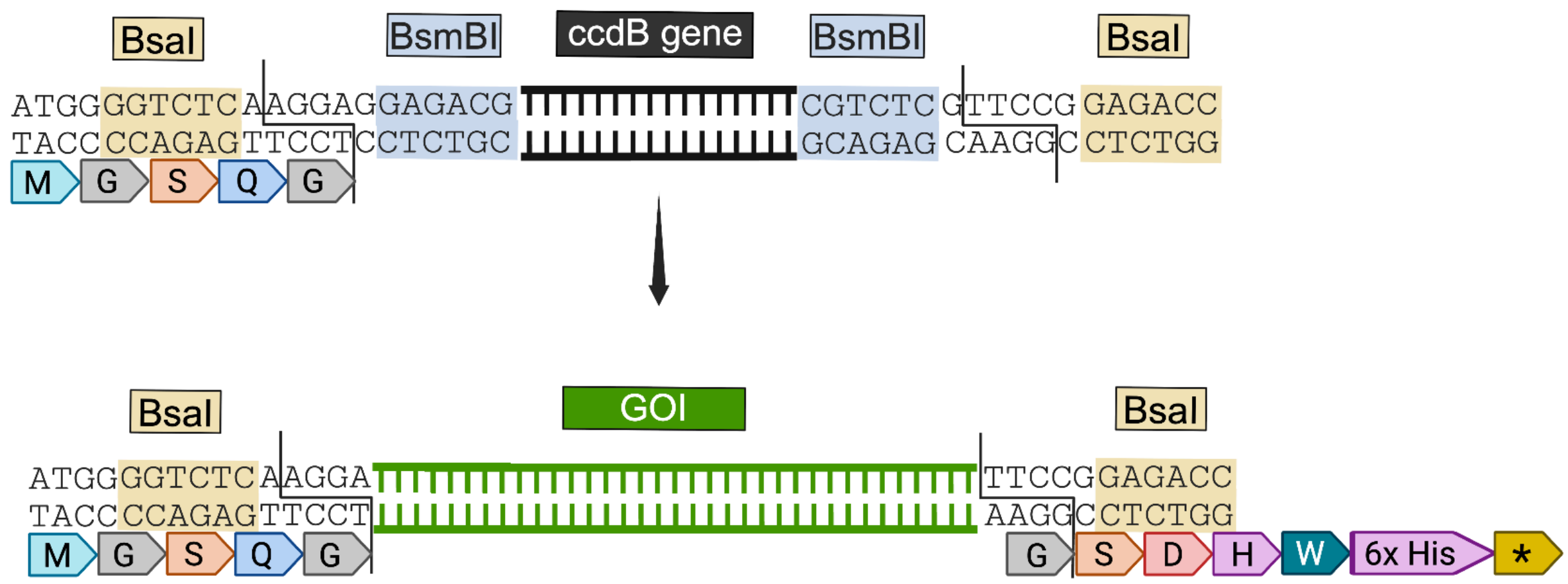

Supplementary Fig. 4. Multi-use cloning strategy for DMX vector. BsmBI enzyme is used to clone GOI library into the DMX vector. BsaI enzyme can be used for barcoding as well as swapping GOI with AGGA/TTCC overhangs into other SAPP entry vectors.

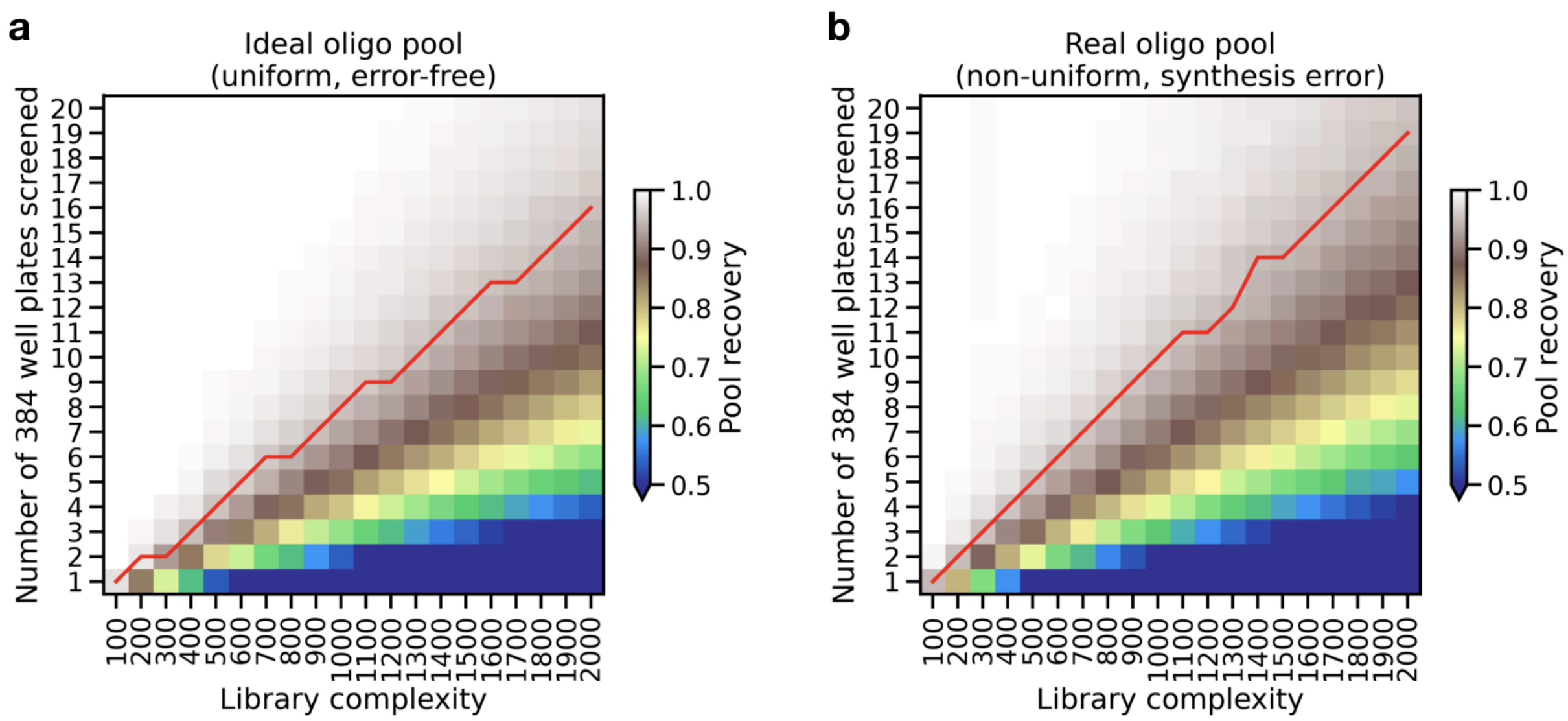

Supplementary Fig. 5. Simulations of expected pool recovery as a function of library complexity and number of screened colonies; (a) assuming an ideal (uniform) pool of error-free oligos, and (b) a biased pool containing oligos with synthesis error. The synthesis error rate used for the simulation was 1/3000 nt, and the oligo abundance bias assumed to be normal and approximating the distribution reported by Twist on their website. The isoline indicating 95% recovery is shown in red.

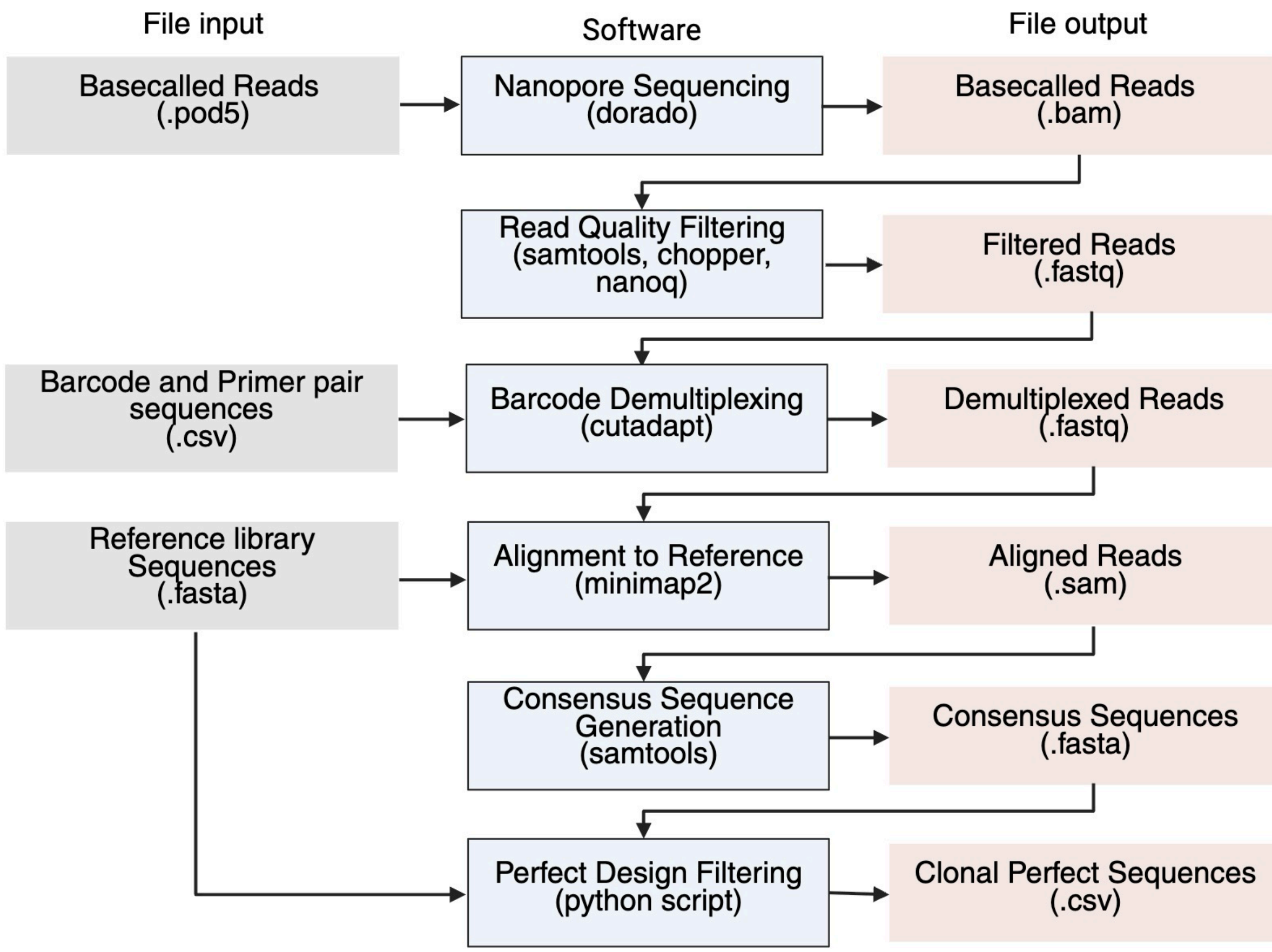

Supplementary Fig. 6. Workflow illustrating bioinformatic analysis to generate clonal perfect sequences from nanopore sequencing reads of pooled barcoded bacterial lysate.

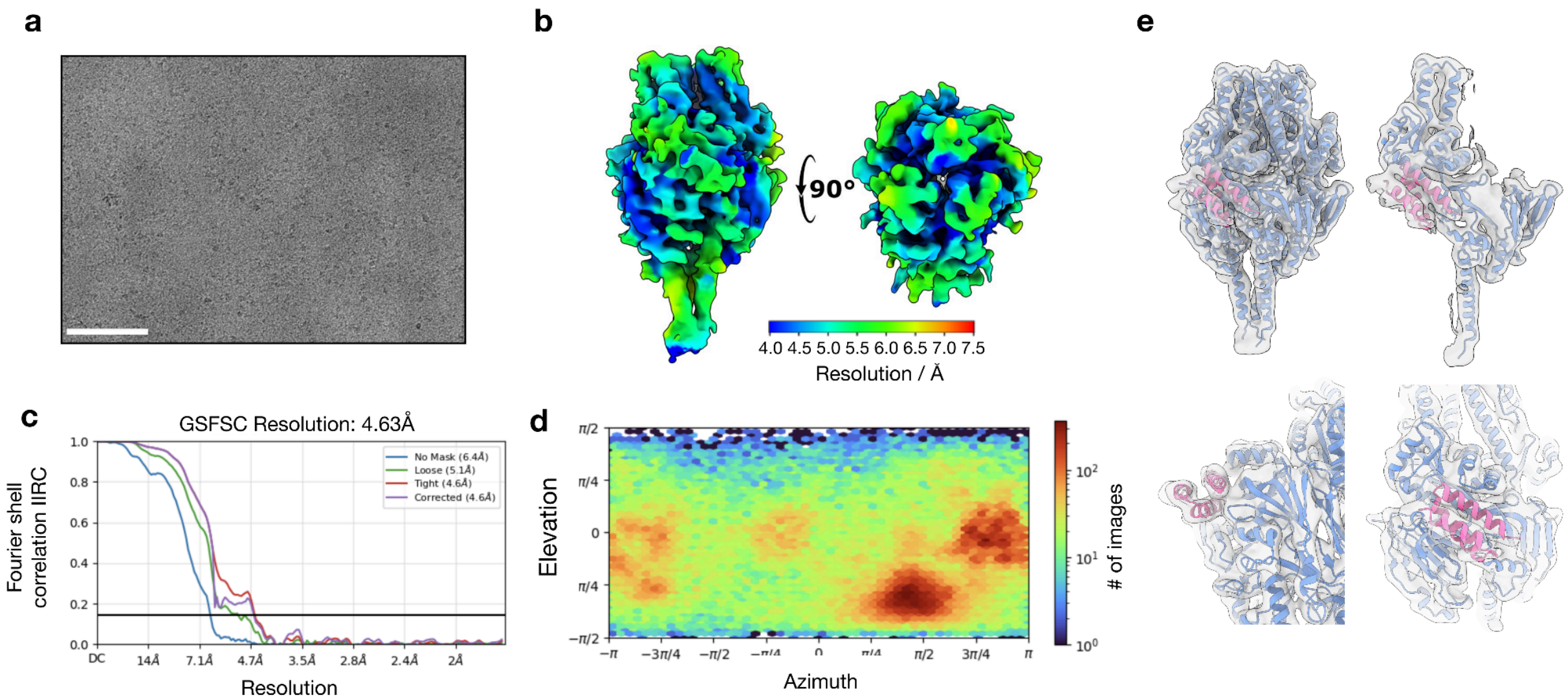

Supplementary Fig. 7. Cryo-EM data processing statistics for cb13 in complex with RSV F. (a) Representative raw cryo-EM micrograph of RSV F bound to cb13. Scale bar: 100 nm. (b) Local resolution map of the RSV F-cb13 complex, with values ranging from ~4 Å in the core to ~6 Å along the periphery. (c) Fourier shell correlation (FSC) curve showing the global resolution estimation with a final resolution of 4.63 Å. (d) Angular distribution plot of particle orientation used in the final 3D reconstruction. (e) Relaxed cryo-EM model docked into the corresponding 3D density map shown from multiple viewing angles to highlight agreement between the model and experimental density. Clockwise starting from upper left: full RSV F-cb13 complex docked into the density map; a single RSV F protomer and bound cb13 minibinder docked into density and zoned to within 5 Å of the structure; zoomed-in view of cb13 bound to RSV F antigenic site III; and the same view rotated by 90° to further demonstrate agreement between the model and density. Density quality was sufficient for confident secondary structure modeling only

Supplementary Tables

Supplementary Table 1. SAPP entry vectors deposited with Addgene. All vectors are compatible with BsaI using AGGA/TTCC overhangs (exception highlighted). All plasmids have a pBR322 origin of replication (medium-copy).

ID	alternate ID	Description	Addgene ID	Antibiotic Resistance	RestrictionEnzyme	GGA_overhang_N	GGA_overhang_C	Link
**cwby0001**	**LM627**	M-AGGA-promoter-ccdb-TTCC-SNAC-HIS	232210	**KanR**	BsaI	AGGA	TTCC	cwby0001
**cwby0002**	**LM670**	MSG-AGGA-promoter-ccdb-TTCC-HIS	232211	**KanR**	BsaI	AGGA	TTCC	cwby0002
**cwby0003**	**LM671**	sfGFP-AGGA-ccdb-TTCC-HIS	232212	**KanR**	BsaI	AGGA	TTCC	cwby0003
**cwby0004**	**LM1369**	cwby template vector to insert arbitrary ccdb containing sequences: pet29-ATG-PaQCI-TAAT_GA	232213	**KanR**	PaqCI	TATG	TAAT	cwby0004
**cwby0005**	**LM1371**	MS-his-AGGA-promoter-ccdb-TTCC	232214	**KanR**	BsaI	AGGA	TTCC	cwby0005
**cwby0006**	**LM1423**	Cwby vector to control entire insert from N-C terminus TATG-promotor-ccdb-TAAT	232215	**KanR**	BsaI	TATG	TAAT	cwby0006
**cwby0007**	**LM1425**	M-AGGA-promoter-ccdb-TTCC-Avitag-HIS	232216	**KanR**	BsaI	AGGA	TTCC	cwby0007

**cwby0008**	**LM1426**	M-AGGA-promoter-ccdb-TTCC-HIS-SpyTag003	232217	**KanR**	BsaI	AGGA	TTCC	cwby0008
**cwby0009**	**LM1487**	his-mScarletI-AGGA-promoter-ccdb-TTCC-his	232218	**KanR**	BsaI	AGGA	TTCC	cwby0009
**cwby0010**	**LM1488**	his-TagGFP2-AGGA-promoter-ccdb-TTCC	232219	**KanR**	BsaI	AGGA	TTCC	cwby0010
**cwby0011**	**LM1489**	his-mTagBFP2-AGGA-promoter-ccdb-TTCC	232220	**KanR**	BsaI	AGGA	TTCC	cwby0011
**cwby0012**	**LM4372**	his-mscarletI3-AGGA-promoter-ccdb-TTCC	232221	**KanR**	BsaI	AGGA	TTCC	cwby0012
**cwby0013**	**LM4373**	his-muGFP-AGGA-promoter-ccdb-TTCC	232222	**KanR**	BsaI	AGGA	TTCC	cwby0013
**cwby0014**	**LM4374**	his-mBaoJin-AGGA-promoter-ccdb-TTCC	232223	**KanR**	BsaI	AGGA	TTCC	cwby0014
**cwby0015**	**LM4376**	his-mhfYFP-AGGA-promoter-ccdb-TTCC	232224	**KanR**	BsaI	AGGA	TTCC	cwby0015
**cwby0016**	**BW1006**	His-lgBiT-AGGA-promoter-ccdb-TTCC	232225	**KanR**	BsaI	AGGA	TTCC	cwby0016
**cwby0017**	**BW1005**	His-GB1-smBiT-AGGA-promoter-ccdb-TTCC	232226	**KanR**	BsaI	AGGA	TTCC	cwby0017
**cwby0018**	**BW1004**	His-smBiT-AGGA-promoter-ccdb-TTCC	232227	**KanR**	BsaI	AGGA	TTCC	cwby0018
**cwby0019**	**BW1003**	AGGA-promoter-ccdb-TTCC-lgBiT-his	232228	**KanR**	BsaI	AGGA	TTCC	cwby0019
**cwby0020**	**BW1002**	AGGA-promoter-ccdb-TTCC-smBiT-GB1-His	232229	**KanR**	BsaI	AGGA	TTCC	cwby0020
**cwby00**	**BW100**	AGGA-promoter-ccdb-TTCC-smBiT-	23223	**KanR**	BsaI	AGGA	TTCC	cwby0021

**21**	**1**	His	0					
**cwby0022**	**BW1008**	M-AGGA-promoter-ccdb-TTCC-SNAC-HIS CarbR	232231	**AmpR / CarbR**	Bsal	AGGA	TTCC	cwby0022
**cwby0023**	**BW1022**	MSG-AGGA-promoter-ccdb-TTCC-HIS CarbR	232232	**AmpR / CarbR**	Bsal	AGGA	TTCC	cwby0023
**cwby0024**	**BW1023**	MS-his-AGGA-promoter-ccdb-TTCC CarbR	232233	**AmpR / CarbR**	Bsal	AGGA	TTCC	cwby0024
**cwby0025**	**BW1024**	TATG-promotor-ccdb-TAAT CarbR	232234	**AmpR / CarbR**	Bsal	AGGA	TTCC	cwby0025
**cwby0026**	**RR001**	AGGA-promoter-ccdb-TTCC-GS-HA LC1_004-SNAC-6xhis	TBD	**KanR**	Bsal	AGGA	TTCC	
**cwby0027**	**RR002**	AGGA-promoter-ccdb-TTCC-GS-HA LC1_008-SNAC-6xhis	TBD	**KanR**	Bsal	AGGA	TTCC	
**cwby0028**	**RR003**	AGGA-promoter-ccdb-TTCC-GS-HA LC2_059-SNAC-6xhis	TBD	**KanR**	Bsal	AGGA	TTCC	
**cwby0029**	**RR004**	AGGA-promoter-ccdb-TTCC-GS-HA LC2_062-SNAC-6xhis	TBD	**KanR**	Bsal	AGGA	TTCC	
**cwby0030**	**RR005**	AGGA-promoter-ccdb-TTCC-GS-HA LC2_063-SNAC-6xhis	TBD	**KanR**	Bsal	AGGA	TTCC	
**cwby0031**	**RR006**	AGGA-promoter-ccdb-TTCC-GS-HA LC2_064-SNAC-6xhis	TBD	**KanR**	Bsal	AGGA	TTCC	
**cwby0032**	**RR007**	AGGA-promoter-ccdb-TTCC-GS-HA LC2_065-SNAC-6xhis	TBD	**KanR**	Bsal	AGGA	TTCC	
**cwby0033**	**RR008**	AGGA-promoter-ccdb-TTCC-GS-HA LC2_067-SNAC-6xhis	TBD	**KanR**	Bsal	AGGA	TTCC	
**cwby0034**	**RR009**	AGGA-promoter-ccdb-TTCC-GS-HA LC2_068-SNAC-6xhis	TBD	**KanR**	Bsal	AGGA	TTCC	

**cwby0035**	**RR010**	AGGA-promoter-ccdb-TTCC-GS-HALC3_104-SNAC-6xhis	TBD	**KanR**	BsaI	AGGA	TTCC	
**cwby0036**	**RR011**	AGGA-promoter-ccdb-TTCC-GS-HALC3_109-SNAC-6xhis	TBD	**KanR**	BsaI	AGGA	TTCC	
**cwby0037**	**RR012**	AGGA-promoter-ccdb-TTCC-GS-HALC3_110-SNAC-6xhis	TBD	**KanR**	BsaI	AGGA	TTCC	
**cwby0038**	**RR013**	AGGA-promoter-ccdb-TTCC-GS-HALC3_114-SNAC-6xhis	TBD	**KanR**	BsaI	AGGA	TTCC	
**cwby0039**	**RR014**	AGGA-promoter-ccdb-TTCC-GS-HALC3_118-SNAC-6xhis	TBD	**KanR**	BsaI	AGGA	TTCC	
**cwby0040**	**RR015**	AGGA-promoter-ccdb-TTCC-GS-HALC3_919-SNAC-6xhis	TBD	**KanR**	BsaI	AGGA	TTCC	
**cwby0041**	**RR016**	AGGA-promoter-ccdb-TTCC-GS-SB175-SNAC-6xhis	TBD	**KanR**	BsaI	AGGA	TTCC	
**cwby0042**	**RR017**	AGGA-promoter-ccdb-TTCC-GS-HALC4_135-SNAC-6xhis	TBD	**KanR**	BsaI	AGGA	TTCC	
**cwby0043**	**RR018**	AGGA-promoter-ccdb-TTCC-GS-HALC4_136-SNAC-6xhis	TBD	**KanR**	BsaI	AGGA	TTCC	
**cwby0044**	**RR019**	AGGA-promoter-ccdb-TTCC-GS-HALC4_140-SNAC-6xhis	TBD	**KanR**	BsaI	AGGA	TTCC	
**cwby0045**	**RR020**	AGGA-promoter-ccdb-TTCC-GS-HALC5_169-SNAC-6xhis	TBD	**KanR**	BsaI	AGGA	TTCC	
**cwby0046**	**RR021**	AGGA-promoter-ccdb-TTCC-GS-HALC5_176-SNAC-6xhis	TBD	**KanR**	BsaI	AGGA	TTCC	
**cwby0047**	**RR022**	AGGA-HALC1_004-GS-promoter-ccdb-TTCC-SNAC-6xhis	TBD	**KanR**	BsaI	AGGA	TTCC	
**cwby00**	**RR023**	AGGA-HALC1_008-GS-promoter-cc	TBD	**KanR**	BsaI	AGGA	TTCC	

**48**		db-TTCC-SNAC-6xhis						
**cwby0049**	**RR024**	AGGA-HALC2_059-GS-promoter-ccdb-TTCC-SNAC-6xhis	TBD	**KanR**	BsaI	AGGA	TTCC	
**cwby0050**	**RR025**	AGGA-HALC2_062-GS-promoter-ccdb-TTCC-SNAC-6xhis	TBD	**KanR**	BsaI	AGGA	TTCC	
**cwby0051**	**RR026**	AGGA-HALC2_063-GS-promoter-ccdb-TTCC-SNAC-6xhis	TBD	**KanR**	BsaI	AGGA	TTCC	
**cwby0052**	**RR027**	AGGA-HALC2_064-GS-promoter-ccdb-TTCC-SNAC-6xhis	TBD	**KanR**	BsaI	AGGA	TTCC	
**cwby0053**	**RR028**	AGGA-HALC2_065-GS-promoter-ccdb-TTCC-SNAC-6xhis	TBD	**KanR**	BsaI	AGGA	TTCC	
**cwby0054**	**RR029**	AGGA-HALC2_067-GS-promoter-ccdb-TTCC-SNAC-6xhis	TBD	**KanR**	BsaI	AGGA	TTCC	
**cwby0055**	**RR030**	AGGA-HALC2_068-GS-promoter-ccdb-TTCC-SNAC-6xhis	TBD	**KanR**	BsaI	AGGA	TTCC	
**cwby0056**	**RR031**	AGGA-HALC3_104-GS-promoter-ccdb-TTCC-SNAC-6xhis	TBD	**KanR**	BsaI	AGGA	TTCC	
**cwby0057**	**RR032**	AGGA-HALC3_109-GS-promoter-ccdb-TTCC-SNAC-6xhis	TBD	**KanR**	BsaI	AGGA	TTCC	
**cwby0058**	**RR033**	AGGA-HALC3_110-GS-promoter-ccdb-TTCC-SNAC-6xhis	TBD	**KanR**	BsaI	AGGA	TTCC	
**cwby0059**	**RR034**	AGGA-HALC3_114-GS-promoter-ccdb-TTCC-SNAC-6xhis	TBD	**KanR**	BsaI	AGGA	TTCC	
**cwby0060**	**RR035**	AGGA-HALC3_118-GS-promoter-ccdb-TTCC-SNAC-6xhis	TBD	**KanR**	BsaI	AGGA	TTCC	
**cwby0061**	**RR036**	AGGA-HALC3_919-GS-promoter-ccdb-TTCC-SNAC-6xhis	TBD	**KanR**	BsaI	AGGA	TTCC	

**cwby0062**	**RR037**	AGGA-SB175-GS-promoter-ccdb-TTCC-SNAC-6xhis	TBD	**KanR**	BsaI	AGGA	TTCC	
**cwby0063**	**RR038**	AGGA-HALC4_135-GS-promoter-ccdb-TTCC-SNAC-6xhis	TBD	**KanR**	BsaI	AGGA	TTCC	
**cwby0064**	**RR039**	AGGA-HALC4_136-GS-promoter-ccdb-TTCC-SNAC-6xhis	TBD	**KanR**	BsaI	AGGA	TTCC	
**cwby0065**	**RR040**	AGGA-HALC4_140-GS-promoter-ccdb-TTCC-SNAC-6xhis	TBD	**KanR**	BsaI	AGGA	TTCC	
**cwby0066**	**RR041**	AGGA-HALC5_169-GS-promoter-ccdb-TTCC-SNAC-6xhis	TBD	**KanR**	BsaI	AGGA	TTCC	
**cwby0067**	**RR042**	AGGA-HALC5_176-GS-promoter-ccdb-TTCC-SNAC-6xhis	TBD	**KanR**	BsaI	AGGA	TTCC	
**cwby0068**	**ME001**	AGGA-promoter-ccdb-TTCC-GS-C2-02802-SNAC-6xhis	TBD	**KanR**	BsaI	AGGA	TTCC	
**cwby0069**	**ME002**	AGGA-promoter-ccdb-TTCC-GS-C4-71-8-SNAC-6xhis	TBD	**KanR**	BsaI	AGGA	TTCC	
**cwby0070**	**ME003**	AGGA-promoter-ccdb-TTCC-GS-C4-81-4-SNAC-6xhis	TBD	**KanR**	BsaI	AGGA	TTCC	
**cwby0071**	**ME004**	AGGA-promoter-ccdb-TTCC-GS-C6-71-9-1-SNAC-6xhis	TBD	**KanR**	BsaI	AGGA	TTCC	
**cwby0072**	**ME005**	AGGA-promoter-ccdb-TTCC-GS-C6-79-10-SNAC-6xhis	TBD	**KanR**	BsaI	AGGA	TTCC	
**cwby0073**	**ME006**	AGGA-promoter-ccdb-TTCC-GS-C8-71-7-1-SNAC-6xhis	TBD	**KanR**	BsaI	AGGA	TTCC	
**cwby0074**	**ME007**	AGGA-promoter-ccdb-TTCC-GS-H6-SNAC-6xhis	TBD	**KanR**	BsaI	AGGA	TTCC	
**cwby00**	**ME008**	AGGA-promoter-ccdb-TTCC-GS-H8	TBD	**KanR**	BsaI	AGGA	TTCC	

**75**		-SNAC-6xhis						
**cwby0076**	**ME009**	AGGA-C2-02802-GS-promoter-ccdb-TTCC-SNAC-6xhis	TBD	**KanR**	BsaI	AGGA	TTCC	
**cwby0077**	**ME010**	AGGA-C4-71-8-GS-promoter-ccdb-TTCC-SNAC-6xhis	TBD	**KanR**	BsaI	AGGA	TTCC	
**cwby0078**	**ME011**	AGGA-C4-81-4-GS-promoter-ccdb-TTCC-SNAC-6xhis	TBD	**KanR**	BsaI	AGGA	TTCC	
**cwby0079**	**ME012**	AGGA-C6-71-9-1-GS-promoter-ccdb-TTCC-SNAC-6xhis	TBD	**KanR**	BsaI	AGGA	TTCC	
**cwby0080**	**ME013**	AGGA-C6-79-10-GS-promoter-ccdb-TTCC-SNAC-6xhis	TBD	**KanR**	BsaI	AGGA	TTCC	
**cwby0081**	**ME014**	AGGA-C8-71-7-1-GS-promoter-ccdb-TTCC-SNAC-6xhis	TBD	**KanR**	BsaI	AGGA	TTCC	
**cwby0082**	**ME015**	AGGA-H6-GS-promoter-ccdb-TTCC-SNAC-6xhis	TBD	**KanR**	BsaI	AGGA	TTCC	
**cwby0083**	**ME016**	AGGA-H8-GS-promoter-ccdb-TTCC-SNAC-6xhis	TBD	**KanR**	BsaI	AGGA	TTCC	
**DMX0001**		M-BsaI_AGGA_BsmBI-promoter-ccdb-BsmBI_TTCC_BsaI_his-W__AMPR	TBD	**AmpR**	BsmBI/BsaI	AGGA	TTCC	
**DMX0002**		M-BsaI_AGGA_BsmBI-promoter-ccdb-BsmBI_TTCC_BsaI_W-his__AMPR	TBD	**AmpR**	BsmBI/BsaI	AGGA	TTCC	

Supplementary Table 2. Cost breakdown for SAPP protocol (starting from gene fragments).

Synthetic DNA	$ / Design	Amount	Units	Usage	$ / Unit	Bulk Amount	Bulk List Price	Supplier	Cat#
eBlocks™ Gene Fragments	21.00	300	bp	1	$ 0.070	1	$ 0.07	IDT	N/A
subtotal:	21.00								
**Cloning**									
BsaI-HF®v2	0.08	1.2	U	1	$ 0.07	5000	$ 331.00	NEB	R3733L
T4 DNA Ligase	0.11	40	U	1	$ 0.00	100000	$ 281.00	NEB	M0202L
Entry vector (e.g. LM0627, Midiprep)	0.13	14	ng	1	$ 0.01	60000	$ 566.00	ZymoResearch	D4201
BL21(DE3) Competent E. Coli	0.92	6	uL	1	$ 0.15	1200	$ 184.00	NEB	C2527I
subtotal:	1.24								
**Protein Expression**									
Terrific broth II	0.04	0.2	g	1	$ 0.18	15000	$ 2,745.10	MP Biomedicals	113046032
Glycerol	0.00	0.016	mL	1	$ 0.13	4000	$ 510.00	MilliporeSigma	G5516-4L
Dextrose (D-Glucose), Anhydrous	0.00	0.002	g	1	$ 0.34	1000	$ 343.50	FisherScientific	D16-1
D-Lactose monohydrate	0.00	0.008	g	1	$ 0.07	1000	$ 72.80	MilliporeSigma	61345-1KG
Kanamycin sulfate	0.00	0.0002	g	1	$ 9.84	100	$ 983.50	FisherScientific	AC450811000
subtotal:	0.04								
**Protein Purification**									
B-PER™	0.35	0.4	mL	1	$ 0.87	500	$ 436.00	ThermoFisher	78248
Benzonase® Nuclease	0.12	10	U	1	$ 0.01	25000	$ 301.00	MilliporeSigma	E1014-25KU
PMSF	0.00	0.0001	g	1	$ 12.80	25	$ 320.00	MilliporeSigma	11359061001

Lysozyme from chicken egg white	0.00	0.00004	g	1	$ 26.60	100	$ 2,660.00	MilliporeSigma	L6876
HisPur™ Ni-NTA Resin	0.02	0.05	mL	20	$ 7.94	500	$ 3,970.00	ThermoFisher	88223
Cytiva Sx 5-150	0.55	1	run	5000	$ 2,745.00	1	$ 2,745.00	Cytiva	29148722
subtotal:	1.04								
**Buffers**									
Trizma® base	0.00	0.017	g	1	$ 0.13	10000	$ 1,300.00	MilliporeSigma	T1503-10KG
NaCl	0.00	0.123	g	1	$ 0.04	2500	$ 95.51	FisherScientific	BP358-212
Imidazole	0.01	0.007	g	1	$ 1.08	1000	$ 1,084.00	MilliporeSigma	1370981000
subtotal:	0.01								
**Plasticware**									
Tips	0.17	4.000	tip	1	$ 0.04	9600	$ 400.00	Opentrons	999-00013
Armadillo PCR plate	0.08	0.010	plate	1	$ 7.64	25	$ 191.00	FisherScientific	AB2396
Deepwell culture plate	0.01	0.010	plate	10	$ 9.22	60	$ 553.00	FisherScientific	278752
Breathable membrane	0.05	0.052	foil	1	$ 0.66	100	$ 65.80	MilliporeSigma	Z763624
96w fritted plate	0.00	0.010	plate	50	$ 19.04	25	$ 476.00	Agilent	200967-100
96w filter plate	0.32	0.010	plate	1	$ 31.96	25	$ 799.00	Agilent	203940-100
96w receiver plate	0.13	0.021	plate	1	$ 6.20	120	$ 743.50	ThermoFisher	249944
384w HPLC collection plate	0.05	0.042	plate	10	$ 11.73	60	$ 704.00	Greiner Bio-One	781270
subtotal:	0.64								
Total cost ($) / design	23.98								
Total cost ($) / 96 designs	2,302.35								

Supplementary Table 3. UMIs used for DMX barcoding.

	BsaI5	overhang5	Seq5	UMI	Seq3	overhang3	BsaI3	Final_Seq_length	BC_num	Sample_num	Final_seq
1	GGTCTCA	TCCT	GTGGGCCATCTTCCTGCGTATCAAA	TATGAGGACGAATCTCCCGCTTATA	GTCTAAAGTATGCGTCGCGGCATGA	GAAC	CGAGACC	97	1	1_1	GGTCTCATCCTGTGGGCCATCTTCCTGCGTATCAAATATGAGGACGAATCTCCCGCTTATAGTCTAAAGTATGCGTCGCGGCATGAGAACCGAGACC
2	GGTCTCA	TCCT	GTGGGCCATCTTCCTGCGTATCAAA	GGTCTTGACAAACGTGTGCTTGTAC	GTCTAAAGTATGCGTCGCGGCATGA	GAAC	CGAGACC	97	1	1_2	GGTCTCATCCTGTGGGCCATCTTCCTGCGTATCAAAGGTCTTGACAAACGTGTGCTTGTACGTCTAAAGTATGCGTCGCGGCATGAGAACCGAGACC
3	GGTCTCA	TCCT	GTGGGCCATCTTCCTGCGTATCAAA	GTTTATCGGGCGTGGTGCTCGCATA	GTCTAAAGTATGCGTCGCGGCATGA	GAAC	CGAGACC	97	1	1_3	GGTCTCATCCTGTGGGCCATCTTCCTGCGTATCAAAGTTTATCGGGCGTGGTGCTCGCATAGTCTAAAGTATGCGTCGCGGCATGAGAACCGAGACC
4	GGTCTCA	TCCT	GTGGGCCATCTTCCTGCGTATCAAA	CCGATGTTGACGGACTAATCCTGAC	GTCTAAAGTATGCGTCGCGGCATGA	GAAC	CGAGACC	97	1	1_4	GGTCTCATCCTGTGGGCCATCTTCCTGCGTATCAAACCGATGTTGACGGACTAATCCTGACGTCTAAAGTATGCGTCGCGGCATGAGAACCGAGACC
5	GGTCTCA	TCCT	GTGGGCCATCTTCCTGCGTATCAAA	TAGTAGTTCAGACGCCGTTAAGCGC	GTCTAAAGTATGCGTCGCGGCATGA	GAAC	CGAGACC	97	1	1_5	GGTCTCATCCTGTGGGCCATCTTCCTGCGTATCAAATAGTAGTTCAGACGCCGTTAAGCGCGTCTAAAGTATGCGTCGCGGCATGAGAACCGAGACC
6	GGTCTCA	TCCT	GTGGGCCATCTTCCTGCGTATCAAA	CCGTACCTAGATACACTCAATTTGT	GTCTAAAGTATGCGTCGCGGCATGA	GAAC	CGAGACC	97	1	1_6	GGTCTCATCCTGTGGGCCATCTTCCTGCGTATCAAACCGTACCTAGATACACTCAATTTGTGTCTAAAGTATGCGTCGCGGCATGAGAACCGAGACC
7	GGTCTCA	TCCT	GTGGGCCATCTTCCTGCGTATCAAA	CTGACGTGTGAGGCGCTAGAGCATA	GTCTAAAGTATGCGTCGCGGCATGA	GAAC	CGAGACC	97	1	1_7	GGTCTCATCCTGTGGGCCATCTTCCTGCGTATCAAACTGACGTGTGAGGCGCTAGAGCATAGTCTAAAGTATGCGTCGCGGCATGAGAACCGAGACC
8	GGTCTCA	TCCT	GTGGGCCATCTTCCTGCGTATCAAA	GGTATGGCACGCCTAATCTGGACAC	GTCTAAAGTATGCGTCGCGGCATGA	GAAC	CGAGACC	97	1	1_8	GGTCTCATCCTGTGGGCCATCTTCCTGCGTATCAAAGGTATGGCACGCCTAATCTGGACACGTCTAAAGTATGCGTCGCGGCATGAGAACCGAGACC

9	GGTCTCA	TCCT	GTGGGCCATCTTCCTGCGTATCAAA	GGATGCATGATCTAGGGCCTCGTCT	GTCTAAAGTATGCGTCGCGGCATGA	GAAC	CGAGACC	97	1	1_9	GGTCTCATCCTGTGGGCCATCTTCCTGCGTATCAAAGGATGCATGATCTAGGGCCTCGTCTGTCTAAAGTATGCGTCGCGGCATGAGAACCGAGACC
10	GGTCTCA	TCCT	GTGGGCCATCTTCCTGCGTATCAAA	GAGGTCTTTCATGCGTATAGTCACA	GTCTAAAGTATGCGTCGCGGCATGA	GAAC	CGAGACC	97	1	1_10	GGTCTCATCCTGTGGGCCATCTTCCTGCGTATCAAAGAGGTCTTTCATGCGTATAGTCACAGTCTAAAGTATGCGTCGCGGCATGAGAACCGAGACC
11	GGTCTCA	TCCT	GTGGGCCATCTTCCTGCGTATCAAA	GATTCAATATGTGTCGTCTATCCTC	GTCTAAAGTATGCGTCGCGGCATGA	GAAC	CGAGACC	97	1	1_11	GGTCTCATCCTGTGGGCCATCTTCCTGCGTATCAAAGATTCAATATGTGTCGTCTATCCTCGTCTAAAGTATGCGTCGCGGCATGAGAACCGAGACC
12	GGTCTCA	TCCT	GTGGGCCATCTTCCTGCGTATCAAA	GGTAACTGCGCATAGTTGGCTCTAT	GTCTAAAGTATGCGTCGCGGCATGA	GAAC	CGAGACC	97	1	1_12	GGTCTCATCCTGTGGGCCATCTTCCTGCGTATCAAAGGTAACTGCGCATAGTTGGCTCTATGTCTAAAGTATGCGTCGCGGCATGAGAACCGAGACC
13	GGTCTCA	TCCT	GTGGGCCATCTTCCTGCGTATCAAA	GCTCTTAAAACTGGTATCACCTGAC	GTCTAAAGTATGCGTCGCGGCATGA	GAAC	CGAGACC	97	1	1_13	GGTCTCATCCTGTGGGCCATCTTCCTGCGTATCAAAGCTCTTAAAACTGGTATCACCTGACGTCTAAAGTATGCGTCGCGGCATGAGAACCGAGACC
14	GGTCTCA	TCCT	GTGGGCCATCTTCCTGCGTATCAAA	GGGTGGTTAGTGATTTGCCCGTCAC	GTCTAAAGTATGCGTCGCGGCATGA	GAAC	CGAGACC	97	1	1_14	GGTCTCATCCTGTGGGCCATCTTCCTGCGTATCAAAGGGTGGTTAGTGATTTGCCCGTCACGTCTAAAGTATGCGTCGCGGCATGAGAACCGAGACC
15	GGTCTCA	TCCT	GTGGGCCATCTTCCTGCGTATCAAA	TAGTTGGTGGGTTTCCCTACCGTGT	GTCTAAAGTATGCGTCGCGGCATGA	GAAC	CGAGACC	97	1	1_15	GGTCTCATCCTGTGGGCCATCTTCCTGCGTATCAAATAGTTGGTGGGTTTCCCTACCGTGTGTCTAAAGTATGCGTCGCGGCATGAGAACCGAGACC
16	GGTCTCA	TCCT	GTGGGCCATCTTCCTGCGTATCAAA	GGTACAGTAAGTGAGAATCCTCTCT	GTCTAAAGTATGCGTCGCGGCATGA	GAAC	CGAGACC	97	1	1_16	GGTCTCATCCTGTGGGCCATCTTCCTGCGTATCAAAGGTACAGTAAGTGAGAATCCTCTCTGTCTAAAGTATGCGTCGCGGCATGAGAACCGAGACC
17	GGTCTCA	TCCT	GTGGGCCATCTTCCTGCGTATCAAA	GGTTCTAAGTTTAGCGTAGCCGGTT	GTCTAAAGTATGCGTCGCGGCATGA	GAAC	CGAGACC	97	1	1_17	GGTCTCATCCTGTGGGCCATCTTCCTGCGTATCAAAGGTTCTAAGTTTAGCGTAGCCGGTTGTCTAAAGTATGCGTCGCGGCATGAGAACCGAGACC

18	GGTCTCA	TCCT	GTGGGCCATCTTCCTGCGTATCAAA	CTTTAGGTGGGTGCGATTGCCAGTT	GTCTAAAGTATGCGTCGCGGCATGA	GAAC	CGAGACC	97	1	1_18	GGTCTCATCCTGTGGGCCATCTTCCTGCGTATCAAACTTTAGGTGGGTGCGATTGCCAGTTGTCTAAAGTATGCGTCGCGGCATGAGAACCGAGACC
19	GGTCTCA	TCCT	GTGGGCCATCTTCCTGCGTATCAAA	TATGTTGTGCCTTACGCCTCGATTA	GTCTAAAGTATGCGTCGCGGCATGA	GAAC	CGAGACC	97	1	1_19	GGTCTCATCCTGTGGGCCATCTTCCTGCGTATCAAATATGTTGTGCCTTACGCCTCGATTAGTCTAAAGTATGCGTCGCGGCATGAGAACCGAGACC
20	GGTCTCA	TCCT	GTGGGCCATCTTCCTGCGTATCAAA	TTAACCGAACTGACGGCCATCAAGG	GTCTAAAGTATGCGTCGCGGCATGA	GAAC	CGAGACC	97	1	1_20	GGTCTCATCCTGTGGGCCATCTTCCTGCGTATCAAATTAACCGAACTGACGGCCATCAAGGGTCTAAAGTATGCGTCGCGGCATGAGAACCGAGACC
21	GGTCTCA	TCCT	GTGGGCCATCTTCCTGCGTATCAAA	GGGTACATGCGCCTTACTCCTTGTG	GTCTAAAGTATGCGTCGCGGCATGA	GAAC	CGAGACC	97	1	1_21	GGTCTCATCCTGTGGGCCATCTTCCTGCGTATCAAAGGGTACATGCGCCTTACTCCTTGTGGTCTAAAGTATGCGTCGCGGCATGAGAACCGAGACC
22	GGTCTCA	TCCT	GTGGGCCATCTTCCTGCGTATCAAA	TTCTATTCTAAGCCGGCGGTCATAT	GTCTAAAGTATGCGTCGCGGCATGA	GAAC	CGAGACC	97	1	1_22	GGTCTCATCCTGTGGGCCATCTTCCTGCGTATCAAATTCTATTCTAAGCCGGCGGTCATATGTCTAAAGTATGCGTCGCGGCATGAGAACCGAGACC
23	GGTCTCA	TCCT	GTGGGCCATCTTCCTGCGTATCAAA	AGAACTATTTCCTGGCTGTTACGCG	GTCTAAAGTATGCGTCGCGGCATGA	GAAC	CGAGACC	97	1	1_23	GGTCTCATCCTGTGGGCCATCTTCCTGCGTATCAAAAGAACTATTTCCTGGCTGTTACGCGGTCTAAAGTATGCGTCGCGGCATGAGAACCGAGACC
24	GGTCTCA	TCCT	GTGGGCCATCTTCCTGCGTATCAAA	TCGGTTTCAAGGATGATCCGCGCTT	GTCTAAAGTATGCGTCGCGGCATGA	GAAC	CGAGACC	97	1	1_24	GGTCTCATCCTGTGGGCCATCTTCCTGCGTATCAAATCGGTTTCAAGGATGATCCGCGCTTGTCTAAAGTATGCGTCGCGGCATGAGAACCGAGACC
25	GGTCTCA	GAAC	CTGGTTGGGATCAGTCGCTTAGTGC	ACTTCTTCTCGGTCGCATGAGGCTG	GAGTGGTCCTTGGGACAGTACCCAG	AAGG	CGAGACC	97	2	2_1	GGTCTCAGAACCTGGTTGGGATCAGTCGCTTAGTGCACTTCTTCTCGGTCGCATGAGGCTGGAGTGGTCCTTGGGACAGTACCCAGAAGGCGAGACC
26	GGTCTCA	GAAC	CTGGTTGGGATCAGTCGCTTAGTGC	GGATACATATACGCTCGTCGGGACT	GAGTGGTCCTTGGGACAGTACCCAG	AAGG	CGAGACC	97	2	2_2	GGTCTCAGAACCTGGTTGGGATCAGTCGCTTAGTGCGGATACATATACGCTCGTCGGGACTGAGTGGTCCTTGGGACAGTACCCAGAAGGCGAGACC

27	GGTCTCA	GAAC	CTGGTTGGGATCAGTCGCTTAGTGC	CTCAGCCTGCCTCGCTTCTGATATT	GAGTGGTCCTTGGGACAGTACCCAG	AAGG	CGAGACC	97	2	2_3	GGTCTCAGAACCTGGTTGGGATCAGTCGCTTAGTGCCTCAGCCTGCCTCGCTTCTGATATTGAGTGGTCCTTGGGACAGTACCCAGAAGGCGAGACC
28	GGTCTCA	GAAC	CTGGTTGGGATCAGTCGCTTAGTGC	GGTAGGGCTACTGTTATCCTCCGTC	GAGTGGTCCTTGGGACAGTACCCAG	AAGG	CGAGACC	97	2	2_4	GGTCTCAGAACCTGGTTGGGATCAGTCGCTTAGTGCGGTAGGGCTACTGTTATCCTCCGTCGAGTGGTCCTTGGGACAGTACCCAGAAGGCGAGACC
29	GGTCTCA	GAAC	CTGGTTGGGATCAGTCGCTTAGTGC	CGTACGGCTGGAGAGCTGTATGTGG	GAGTGGTCCTTGGGACAGTACCCAG	AAGG	CGAGACC	97	2	2_5	GGTCTCAGAACCTGGTTGGGATCAGTCGCTTAGTGCCGTACGGCTGGAGAGCTGTATGTGGGAGTGGTCCTTGGGACAGTACCCAGAAGGCGAGACC
30	GGTCTCA	GAAC	CTGGTTGGGATCAGTCGCTTAGTGC	ACAGGTTGTATTACTTCGCGCCTTG	GAGTGGTCCTTGGGACAGTACCCAG	AAGG	CGAGACC	97	2	2_6	GGTCTCAGAACCTGGTTGGGATCAGTCGCTTAGTGCACAGGTTGTATTACTTCGCGCCTTGGAGTGGTCCTTGGGACAGTACCCAGAAGGCGAGACC
31	GGTCTCA	GAAC	CTGGTTGGGATCAGTCGCTTAGTGC	CTGGGCTCATTACAAGTGTTGCATA	GAGTGGTCCTTGGGACAGTACCCAG	AAGG	CGAGACC	97	2	2_7	GGTCTCAGAACCTGGTTGGGATCAGTCGCTTAGTGCCTGGGCTCATTACAAGTGTTGCATAGAGTGGTCCTTGGGACAGTACCCAGAAGGCGAGACC
32	GGTCTCA	GAAC	CTGGTTGGGATCAGTCGCTTAGTGC	CTAAGTGGCGCCGATTGTTTGTCCA	GAGTGGTCCTTGGGACAGTACCCAG	AAGG	CGAGACC	97	2	2_8	GGTCTCAGAACCTGGTTGGGATCAGTCGCTTAGTGCCTAAGTGGCGCCGATTGTTTGTCCAGAGTGGTCCTTGGGACAGTACCCAGAAGGCGAGACC
33	GGTCTCA	GAAC	CTGGTTGGGATCAGTCGCTTAGTGC	GTATATTTTGCTCCCGGCGACGAGA	GAGTGGTCCTTGGGACAGTACCCAG	AAGG	CGAGACC	97	2	2_9	GGTCTCAGAACCTGGTTGGGATCAGTCGCTTAGTGCGTATATTTTGCTCCCGGCGACGAGAGAGTGGTCCTTGGGACAGTACCCAGAAGGCGAGACC
34	GGTCTCA	GAAC	CTGGTTGGGATCAGTCGCTTAGTGC	GCAATTTGCGCTTGTTCGGCATAGC	GAGTGGTCCTTGGGACAGTACCCAG	AAGG	CGAGACC	97	2	2_10	GGTCTCAGAACCTGGTTGGGATCAGTCGCTTAGTGCGCAATTTGCGCTTGTTCGGCATAGCGAGTGGTCCTTGGGACAGTACCCAGAAGGCGAGACC
35	GGTCTCA	GAAC	CTGGTTGGGATCAGTCGCTTAGTGC	GAGTCGAATATCCACCACCGTATGG	GAGTGGTCCTTGGGACAGTACCCAG	AAGG	CGAGACC	97	2	2_11	GGTCTCAGAACCTGGTTGGGATCAGTCGCTTAGTGCGAGTCGAATATCCACCACCGTATGGGAGTGGTCCTTGGGACAGTACCCAGAAGGCGAGACC

36	GGTCTCA	GAAC	CTGGTTGGGATCAGTCGCTTAGTGC	TTGTGGTTTGGGTCCTCAGAGGAGA	GAGTGGTCCTTGGGACAGTACCCAG	AAGG	CGAGACC	97	2	2_12	GGTCTCAGAACCTGGTTGGGATCAGTCGCTTAGTGCTTGTGGTTTGGGTCCTCAGAGGAGAGAGTGGTCCTTGGGACAGTACCCAGAAGGCGAGACC
37	GGTCTCA	GAAC	CTGGTTGGGATCAGTCGCTTAGTGC	CCGGCGCAGAAGTTTGAACGAAAAG	GAGTGGTCCTTGGGACAGTACCCAG	AAGG	CGAGACC	97	2	2_13	GGTCTCAGAACCTGGTTGGGATCAGTCGCTTAGTGCCCGGCGCAGAAGTTTGAACGAAAAGGAGTGGTCCTTGGGACAGTACCCAGAAGGCGAGACC
38	GGTCTCA	GAAC	CTGGTTGGGATCAGTCGCTTAGTGC	ATGCACTATTTTACGTATCCCGTGC	GAGTGGTCCTTGGGACAGTACCCAG	AAGG	CGAGACC	97	2	2_14	GGTCTCAGAACCTGGTTGGGATCAGTCGCTTAGTGCATGCACTATTTTACGTATCCCGTGCGAGTGGTCCTTGGGACAGTACCCAGAAGGCGAGACC
39	GGTCTCA	GAAC	CTGGTTGGGATCAGTCGCTTAGTGC	GATAGGGTGACTGCTTTCGCGTACA	GAGTGGTCCTTGGGACAGTACCCAG	AAGG	CGAGACC	97	2	2_15	GGTCTCAGAACCTGGTTGGGATCAGTCGCTTAGTGCGATAGGGTGACTGCTTTCGCGTACAGAGTGGTCCTTGGGACAGTACCCAGAAGGCGAGACC
40	GGTCTCA	GAAC	CTGGTTGGGATCAGTCGCTTAGTGC	TATCTGGTAGACATCTCGGCACAGA	GAGTGGTCCTTGGGACAGTACCCAG	AAGG	CGAGACC	97	2	2_16	GGTCTCAGAACCTGGTTGGGATCAGTCGCTTAGTGCTATCTGGTAGACATCTCGGCACAGAGAGTGGTCCTTGGGACAGTACCCAGAAGGCGAGACC
41	GGTCTCA	GAAC	CTGGTTGGGATCAGTCGCTTAGTGC	TAGTTCTGGCTATACACACTTCGGG	GAGTGGTCCTTGGGACAGTACCCAG	AAGG	CGAGACC	97	2	2_17	GGTCTCAGAACCTGGTTGGGATCAGTCGCTTAGTGCTAGTTCTGGCTATACACACTTCGGGGAGTGGTCCTTGGGACAGTACCCAGAAGGCGAGACC
42	GGTCTCA	GAAC	CTGGTTGGGATCAGTCGCTTAGTGC	GCATAGAGTTACCCGATGGATTCGA	GAGTGGTCCTTGGGACAGTACCCAG	AAGG	CGAGACC	97	2	2_18	GGTCTCAGAACCTGGTTGGGATCAGTCGCTTAGTGCGCATAGAGTTACCCGATGGATTCGAGAGTGGTCCTTGGGACAGTACCCAGAAGGCGAGACC
43	GGTCTCA	GAAC	CTGGTTGGGATCAGTCGCTTAGTGC	GTTCATGGTACAGGCTTCTTTACGG	GAGTGGTCCTTGGGACAGTACCCAG	AAGG	CGAGACC	97	2	2_19	GGTCTCAGAACCTGGTTGGGATCAGTCGCTTAGTGCGTTCATGGTACAGGCTTCTTTACGGGAGTGGTCCTTGGGACAGTACCCAGAAGGCGAGACC
44	GGTCTCA	GAAC	CTGGTTGGGATCAGTCGCTTAGTGC	CGATCTCGGGCCTGGGTTTTGAGTA	GAGTGGTCCTTGGGACAGTACCCAG	AAGG	CGAGACC	97	2	2_20	GGTCTCAGAACCTGGTTGGGATCAGTCGCTTAGTGCCGATCTCGGGCCTGGGTTTTGAGTAGAGTGGTCCTTGGGACAGTACCCAGAAGGCGAGACC

45	GGTCTCA	GAAC	CTGGTTGGGATCAGTCGCTTAGTGC	ATTATTCGTGACCCAACTCATCAGG	GAGTGGTCCTTGGGACAGTACCCAG	AAGG	CGAGACC	97	2	2_21	GGTCTCAGAACCTGGTTGGGATCAGTCGCTTAGTGCATTATTCGTGACCCAACTCATCAGGGAGTGGTCCTTGGGACAGTACCCAGAAGGCGAGACC
46	GGTCTCA	GAAC	CTGGTTGGGATCAGTCGCTTAGTGC	CTGAATGGTGAATAATGCGTTCGCC	GAGTGGTCCTTGGGACAGTACCCAG	AAGG	CGAGACC	97	2	2_22	GGTCTCAGAACCTGGTTGGGATCAGTCGCTTAGTGCCTGAATGGTGAATAATGCGTTCGCCGAGTGGTCCTTGGGACAGTACCCAGAAGGCGAGACC
47	GGTCTCA	GAAC	CTGGTTGGGATCAGTCGCTTAGTGC	AGAAAGTCTTGGATACACGGCCGGG	GAGTGGTCCTTGGGACAGTACCCAG	AAGG	CGAGACC	97	2	2_23	GGTCTCAGAACCTGGTTGGGATCAGTCGCTTAGTGCAGAAAGTCTTGGATACACGGCCGGGGAGTGGTCCTTGGGACAGTACCCAGAAGGCGAGACC
48	GGTCTCA	GAAC	CTGGTTGGGATCAGTCGCTTAGTGC	GTGTGTTCCTATGCACAATTTCATA	GAGTGGTCCTTGGGACAGTACCCAG	AAGG	CGAGACC	97	2	2_24	GGTCTCAGAACCTGGTTGGGATCAGTCGCTTAGTGCGTGTGTTCCTATGCACAATTTCATAGAGTGGTCCTTGGGACAGTACCCAGAAGGCGAGACC
49	GGTCTCA	AAGG	CGACACCGAACGTGCGACAAAACTA	TCGTTTGGAGCCGTTCACACATGAA	TGGATAAACCGGCTAGGTCGCAGAG	CTGA	CGAGACC	97	3	3_1	GGTCTCAAAGGCGACACCGAACGTGCGACAAAACTATCGTTTGGAGCCGTTCACACATGAATGGATAAACCGGCTAGGTCGCAGAGCTGACGAGACC
50	GGTCTCA	AAGG	CGACACCGAACGTGCGACAAAACTA	CTGATCAACTTGCGCCCAGCGTTAT	TGGATAAACCGGCTAGGTCGCAGAG	CTGA	CGAGACC	97	3	3_2	GGTCTCAAAGGCGACACCGAACGTGCGACAAAACTACTGATCAACTTGCGCCCAGCGTTATTGGATAAACCGGCTAGGTCGCAGAGCTGACGAGACC
51	GGTCTCA	AAGG	CGACACCGAACGTGCGACAAAACTA	GACGATGTTGCCTGTTTTGATACGA	TGGATAAACCGGCTAGGTCGCAGAG	CTGA	CGAGACC	97	3	3_3	GGTCTCAAAGGCGACACCGAACGTGCGACAAAACTAGACGATGTTGCCTGTTTTGATACGATGGATAAACCGGCTAGGTCGCAGAGCTGACGAGACC
52	GGTCTCA	AAGG	CGACACCGAACGTGCGACAAAACTA	GGGTAGTCGTGAGGTGAACTCTTCC	TGGATAAACCGGCTAGGTCGCAGAG	CTGA	CGAGACC	97	3	3_4	GGTCTCAAAGGCGACACCGAACGTGCGACAAAACTAGGGTAGTCGTGAGGTGAACTCTTCCTGGATAAACCGGCTAGGTCGCAGAGCTGACGAGACC
53	GGTCTCA	AAGG	CGACACCGAACGTGCGACAAAACTA	AGCCATTTTACGATTCTATTCGATG	TGGATAAACCGGCTAGGTCGCAGAG	CTGA	CGAGACC	97	3	3_5	GGTCTCAAAGGCGACACCGAACGTGCGACAAAACTAAGCCATTTTACGATTCTATTCGATGTGGATAAACCGGCTAGGTCGCAGAGCTGACGAGACC

54	GGTCTCA	AAGG	CGACA<br>CCGAA<br>CGTGC<br>GACAA<br>AACTA	GTGGTTT<br>ATATAAT<br>CCCACCT<br>CCTA	TGGATAAAC<br>CGGCTAGGT<br>CGCAGAG	CTGA	CGAGACC	97	3	3_6	GGTCTCAAAGGCGACACCGAACGTGCGACAAAACTAGTGGT<br>TTATATAATCCCACCTCCTATGGATAAACCGGCTAGGTCGC<br>AGAGCTGACGAGACC
55	GGTCTCA	AAGG	CGACA<br>CCGAA<br>CGTGC<br>GACAA<br>AACTA	GCGAAGA<br>ACATCCC<br>GGCATTT<br>CATG	TGGATAAAC<br>CGGCTAGGT<br>CGCAGAG	CTGA	CGAGACC	97	3	3_7	GGTCTCAAAGGCGACACCGAACGTGCGACAAAACTAGCGAA<br>GAACATCCCGGCATTTCATGTGGATAAACCGGCTAGGTCGC<br>AGAGCTGACGAGACC
56	GGTCTCA	AAGG	CGACA<br>CCGAA<br>CGTGC<br>GACAA<br>AACTA	GCTGGGA<br>CAATGCC<br>GAAAACT<br>CTTC	TGGATAAAC<br>CGGCTAGGT<br>CGCAGAG	CTGA	CGAGACC	97	3	3_8	GGTCTCAAAGGCGACACCGAACGTGCGACAAAACTAGCTGG<br>GACAATGCCGAAAACTCTTCTGGATAAACCGGCTAGGTCGC<br>AGAGCTGACGAGACC
57	GGTCTCA	AAGG	CGACA<br>CCGAA<br>CGTGC<br>GACAA<br>AACTA	ATTCCGT<br>ACCAACC<br>CGCGTCT<br>TAGA	TGGATAAAC<br>CGGCTAGGT<br>CGCAGAG	CTGA	CGAGACC	97	3	3_9	GGTCTCAAAGGCGACACCGAACGTGCGACAAAACTAATTCC<br>GTACCAACCCGCGTCTTAGATGGATAAACCGGCTAGGTCGC<br>AGAGCTGACGAGACC
58	GGTCTCA	AAGG	CGACA<br>CCGAA<br>CGTGC<br>GACAA<br>AACTA	GCTGAGG<br>AAGCCCA<br>ATGTTCA<br>GTAC	TGGATAAAC<br>CGGCTAGGT<br>CGCAGAG	CTGA	CGAGACC	97	3	3_10	GGTCTCAAAGGCGACACCGAACGTGCGACAAAACTAGCTGA<br>GGAAGCCCAATGTTCAGTACTGGATAAACCGGCTAGGTCGC<br>AGAGCTGACGAGACC
59	GGTCTCA	AAGG	CGACA<br>CCGAA<br>CGTGC<br>GACAA<br>AACTA	GTAACCT<br>TAGCACG<br>CCGGAGT<br>GGAG	TGGATAAAC<br>CGGCTAGGT<br>CGCAGAG	CTGA	CGAGACC	97	3	3_11	GGTCTCAAAGGCGACACCGAACGTGCGACAAAACTAGTAAC<br>CTTAGCACGCCGGAGTGGAGTGGATAAACCGGCTAGGTCGC<br>AGAGCTGACGAGACC
60	GGTCTCA	AAGG	CGACA<br>CCGAA<br>CGTGC<br>GACAA<br>AACTA	GCGATTA<br>GTTCTGT<br>TGCTAAA<br>CCAG	TGGATAAAC<br>CGGCTAGGT<br>CGCAGAG	CTGA	CGAGACC	97	3	3_12	GGTCTCAAAGGCGACACCGAACGTGCGACAAAACTAGCGAT<br>TAGTTCTGTTGCTAAACCAGTGGATAAACCGGCTAGGTCGC<br>AGAGCTGACGAGACC
61	GGTCTCA	AAGG	CGACA<br>CCGAA<br>CGTGC<br>GACAA<br>AACTA	CTTAAAG<br>GTGATTC<br>ACACGTG<br>TGCC	TGGATAAAC<br>CGGCTAGGT<br>CGCAGAG	CTGA	CGAGACC	97	3	3_13	GGTCTCAAAGGCGACACCGAACGTGCGACAAAACTACTTAA<br>AGGTGATTCACACGTGTGCCTGGATAAACCGGCTAGGTCGC<br>AGAGCTGACGAGACC
62	GGTCTCA	AAGG	CGACA<br>CCGAA<br>CGTGC<br>GACAA<br>AACTA	ATACTTA<br>TTCCGCT<br>CATTGCA<br>CAGG	TGGATAAAC<br>CGGCTAGGT<br>CGCAGAG	CTGA	CGAGACC	97	3	3_14	GGTCTCAAAGGCGACACCGAACGTGCGACAAAACTAATACT<br>TATTCCGCTCATTGCACAGGTGGATAAACCGGCTAGGTCGC<br>AGAGCTGACGAGACC

63	GGTCTCA	AAGG	CGACA<br>CCGAA<br>CGTGC<br>GACAA<br>AACTA	TGAAACC<br>AATTTCA<br>CCTCAGC<br>GGCG	TGGATAAAC<br>CGGCTAGGT<br>CGCAGAG	CTGA	CGAGACC	97	3	3_15	GGTCTCAAAGGCGACACCGAACGTGCGACAAAACTATGAAA<br>CCAATTTCACCTCAGCGGCGTGGATAAACCGGCTAGGTCGC<br>AGAGCTGACGAGACC
64	GGTCTCA	AAGG	CGACA<br>CCGAA<br>CGTGC<br>GACAA<br>AACTA	CGACCAC<br>TCGCCTC<br>CCGTTAT<br>GATC	TGGATAAAC<br>CGGCTAGGT<br>CGCAGAG	CTGA	CGAGACC	97	3	3_16	GGTCTCAAAGGCGACACCGAACGTGCGACAAAACTACGACC<br>ACTCGCCTCCCGTTATGATCTGGATAAACCGGCTAGGTCGC<br>AGAGCTGACGAGACC
65	GGTCTCA	AAGG	CGACA<br>CCGAA<br>CGTGC<br>GACAA<br>AACTA	GTTAATG<br>CTGTTTA<br>GCGTAAC<br>CTCG	TGGATAAAC<br>CGGCTAGGT<br>CGCAGAG	CTGA	CGAGACC	97	3	3_17	GGTCTCAAAGGCGACACCGAACGTGCGACAAAACTAGTTAA<br>TGCTGTTTAGCGTAACCTCGTGGATAAACCGGCTAGGTCGC<br>AGAGCTGACGAGACC
66	GGTCTCA	AAGG	CGACA<br>CCGAA<br>CGTGC<br>GACAA<br>AACTA	GTCTAAT<br>ATGGCGT<br>AGCTCAA<br>CGCG	TGGATAAAC<br>CGGCTAGGT<br>CGCAGAG	CTGA	CGAGACC	97	3	3_18	GGTCTCAAAGGCGACACCGAACGTGCGACAAAACTAGTCTA<br>ATATGGCGTAGCTCAACGCGTGGATAAACCGGCTAGGTCGC<br>AGAGCTGACGAGACC
67	GGTCTCA	AAGG	CGACA<br>CCGAA<br>CGTGC<br>GACAA<br>AACTA	AGTAAAT<br>GGCCTGA<br>CCGGAGT<br>TGGT	TGGATAAAC<br>CGGCTAGGT<br>CGCAGAG	CTGA	CGAGACC	97	3	3_19	GGTCTCAAAGGCGACACCGAACGTGCGACAAAACTAAGTAA<br>ATGGCCTGACCGGAGTTGGTTGGATAAACCGGCTAGGTCGC<br>AGAGCTGACGAGACC
68	GGTCTCA	AAGG	CGACA<br>CCGAA<br>CGTGC<br>GACAA<br>AACTA	ATGTGAA<br>ATGAGGT<br>CTACCCT<br>GTCA	TGGATAAAC<br>CGGCTAGGT<br>CGCAGAG	CTGA	CGAGACC	97	3	3_20	GGTCTCAAAGGCGACACCGAACGTGCGACAAAACTAATGTG<br>AAATGAGGTCTACCCTGTCATGGATAAACCGGCTAGGTCGC<br>AGAGCTGACGAGACC
69	GGTCTCA	AAGG	CGACA<br>CCGAA<br>CGTGC<br>GACAA<br>AACTA	GTGTGTC<br>CTACCTA<br>CGCGAGG<br>AATT	TGGATAAAC<br>CGGCTAGGT<br>CGCAGAG	CTGA	CGAGACC	97	3	3_21	GGTCTCAAAGGCGACACCGAACGTGCGACAAAACTAGTGTG<br>TCCTACCTACGCGAGGAATTTGGATAAACCGGCTAGGTCGC<br>AGAGCTGACGAGACC
70	GGTCTCA	AAGG	CGACA<br>CCGAA<br>CGTGC<br>GACAA<br>AACTA	TTTGGAC<br>GATATAC<br>AGGACCC<br>GGTG	TGGATAAAC<br>CGGCTAGGT<br>CGCAGAG	CTGA	CGAGACC	97	3	3_22	GGTCTCAAAGGCGACACCGAACGTGCGACAAAACTATTTGG<br>ACGATATACAGGACCCGGTGTGGATAAACCGGCTAGGTCGC<br>AGAGCTGACGAGACC
71	GGTCTCA	AAGG	CGACA<br>CCGAA<br>CGTGC<br>GACAA<br>AACTA	AAGCGTA<br>CCAACTA<br>CCTCCGA<br>GTCT	TGGATAAAC<br>CGGCTAGGT<br>CGCAGAG	CTGA	CGAGACC	97	3	3_23	GGTCTCAAAGGCGACACCGAACGTGCGACAAAACTAAAGCG<br>TACCAACTACCTCCGAGTCTTGGATAAACCGGCTAGGTCGC<br>AGAGCTGACGAGACC

72	GGTCTCA	AAGG	CGACA<br>CCGAA<br>CGTGC<br>GACAA<br>AACTA	TTGTGTC<br>GGCCACA<br>GGTACGA<br>AAAC	TGGATAAAC<br>CGGCTAGGT<br>CGCAGAG	CTGA	CGAGACC	97	3	3_24	GGTCTCAAAGGCGACACCGAACGTGCGACAAAACTATTGTG<br>TCGGCCACAGGTACGAAAACTGGATAAACCGGCTAGGTCGC<br>AGAGCTGACGAGACC
73	GGTCTCA	CTGA	TTTAT<br>TCAGG<br>GCACT<br>ACCCG<br>GAGCT	TAGCGAT<br>TATGTTC<br>CCGTTTT<br>AAGC	ATCGGTGAC<br>GGCGATTCT<br>CACATTT	GGAA	CGAGACC	97	4	4_1	GGTCTCACTGATTTATTCAGGGCACTACCCGGAGCTTAGCG<br>ATTATGTTCCCGTTTTAAGCATCGGTGACGGCGATTCTCAC<br>ATTTGGAACGAGACC
74	GGTCTCA	CTGA	TTTAT<br>TCAGG<br>GCACT<br>ACCCG<br>GAGCT	GCTATGG<br>CTCGTGA<br>AGTGAAA<br>CGAA	ATCGGTGAC<br>GGCGATTCT<br>CACATTT	GGAA	CGAGACC	97	4	4_2	GGTCTCACTGATTTATTCAGGGCACTACCCGGAGCTGCTAT<br>GGCTCGTGAAGTGAAACGAAATCGGTGACGGCGATTCTCAC<br>ATTTGGAACGAGACC
75	GGTCTCA	CTGA	TTTAT<br>TCAGG<br>GCACT<br>ACCCG<br>GAGCT	ACCGTAT<br>GGGCGTC<br>ATAAAGG<br>GAGC	ATCGGTGAC<br>GGCGATTCT<br>CACATTT	GGAA	CGAGACC	97	4	4_3	GGTCTCACTGATTTATTCAGGGCACTACCCGGAGCTACCGT<br>ATGGGCGTCATAAAGGGAGCATCGGTGACGGCGATTCTCAC<br>ATTTGGAACGAGACC
76	GGTCTCA	CTGA	TTTAT<br>TCAGG<br>GCACT<br>ACCCG<br>GAGCT	GTAGTTT<br>TCTCATT<br>TCGCGCC<br>TCGG	ATCGGTGAC<br>GGCGATTCT<br>CACATTT	GGAA	CGAGACC	97	4	4_4	GGTCTCACTGATTTATTCAGGGCACTACCCGGAGCTGTAGT<br>TTTCTCATTTCGCGCCTCGGATCGGTGACGGCGATTCTCAC<br>ATTTGGAACGAGACC
77	GGTCTCA	CTGA	TTTAT<br>TCAGG<br>GCACT<br>ACCCG<br>GAGCT	TGGGATG<br>GCGTCGT<br>CTTAGCG<br>TTAT	ATCGGTGAC<br>GGCGATTCT<br>CACATTT	GGAA	CGAGACC	97	4	4_5	GGTCTCACTGATTTATTCAGGGCACTACCCGGAGCTTGGGA<br>TGGCGTCGTCTTAGCGTTATATCGGTGACGGCGATTCTCAC<br>ATTTGGAACGAGACC
78	GGTCTCA	CTGA	TTTAT<br>TCAGG<br>GCACT<br>ACCCG<br>GAGCT	GGCGATA<br>TAGGAGA<br>GTCCGCG<br>CTAA	ATCGGTGAC<br>GGCGATTCT<br>CACATTT	GGAA	CGAGACC	97	4	4_6	GGTCTCACTGATTTATTCAGGGCACTACCCGGAGCTGGCGA<br>TATAGGAGAGTCCGCGCTAAATCGGTGACGGCGATTCTCAC<br>ATTTGGAACGAGACC
79	GGTCTCA	CTGA	TTTAT<br>TCAGG<br>GCACT<br>ACCCG<br>GAGCT	TTCATAT<br>TTAGGTC<br>GTCCGCT<br>CTTA	ATCGGTGAC<br>GGCGATTCT<br>CACATTT	GGAA	CGAGACC	97	4	4_7	GGTCTCACTGATTTATTCAGGGCACTACCCGGAGCTTTCAT<br>ATTTAGGTCGTCCGCTCTTAATCGGTGACGGCGATTCTCAC<br>ATTTGGAACGAGACC
80	GGTCTCA	CTGA	TTTAT<br>TCAGG<br>GCACT<br>ACCCG<br>GAGCT	GGTCGGT<br>CAATTCC<br>CTCCTAA<br>GGAG	ATCGGTGAC<br>GGCGATTCT<br>CACATTT	GGAA	CGAGACC	97	4	4_8	GGTCTCACTGATTTATTCAGGGCACTACCCGGAGCTGGTCG<br>GTCAATTCCCTCCTAAGGAGATCGGTGACGGCGATTCTCAC<br>ATTTGGAACGAGACC

81	GGTCTCA	CTGA	TTTAT TCAGG GCACT ACCCG GAGCT	GCTAACT GGAACGT CCCGGAA TTGC	ATCGGTGAC GGCGATTCT CACATTT	GGAA	CGAGACC	97	4	4_9	GGTCTCACTGATTTATTCAGGGCACTACCCGGAGCTGCTAA CTGGAACGTCCCGGAATTGCATCGGTGACGGCGATTCTCAC ATTTGGAACGAGACC
82	GGTCTCA	CTGA	TTTAT TCAGG GCACT ACCCG GAGCT	AGTCTTT CACAACC CTACGGT GACT	ATCGGTGAC GGCGATTCT CACATTT	GGAA	CGAGACC	97	4	4_10	GGTCTCACTGATTTATTCAGGGCACTACCCGGAGCTAGTCT TTCACAACCCTACGGTGACTATCGGTGACGGCGATTCTCAC ATTTGGAACGAGACC
83	GGTCTCA	CTGA	TTTAT TCAGG GCACT ACCCG GAGCT	GGTGACT TATGAAC CTTTGCG CATT	ATCGGTGAC GGCGATTCT CACATTT	GGAA	CGAGACC	97	4	4_11	GGTCTCACTGATTTATTCAGGGCACTACCCGGAGCTGGTGA CTTATGAACCTTTGCGCATTATCGGTGACGGCGATTCTCAC ATTTGGAACGAGACC
84	GGTCTCA	CTGA	TTTAT TCAGG GCACT ACCCG GAGCT	TATTCGA GGATGGA TTCGGGC TCCT	ATCGGTGAC GGCGATTCT CACATTT	GGAA	CGAGACC	97	4	4_12	GGTCTCACTGATTTATTCAGGGCACTACCCGGAGCTTATTC GAGGATGGATTCGGGCTCCTATCGGTGACGGCGATTCTCAC ATTTGGAACGAGACC
85	GGTCTCA	CTGA	TTTAT TCAGG GCACT ACCCG GAGCT	TCATTCG CTAAATC AGTCGAG TTAG	ATCGGTGAC GGCGATTCT CACATTT	GGAA	CGAGACC	97	4	4_13	GGTCTCACTGATTTATTCAGGGCACTACCCGGAGCTTCATT CGCTAAATCAGTCGAGTTAGATCGGTGACGGCGATTCTCAC ATTTGGAACGAGACC
86	GGTCTCA	CTGA	TTTAT TCAGG GCACT ACCCG GAGCT	AACGGGA GTCCGAG GTCGTAC CTAG	ATCGGTGAC GGCGATTCT CACATTT	GGAA	CGAGACC	97	4	4_14	GGTCTCACTGATTTATTCAGGGCACTACCCGGAGCTAACGG GAGTCCGAGGTCGTACCTAGATCGGTGACGGCGATTCTCAC ATTTGGAACGAGACC
87	GGTCTCA	CTGA	TTTAT TCAGG GCACT ACCCG GAGCT	GGATGGG AACCCAA CACCTGT TCAT	ATCGGTGAC GGCGATTCT CACATTT	GGAA	CGAGACC	97	4	4_15	GGTCTCACTGATTTATTCAGGGCACTACCCGGAGCTGGATG GGAACCCAACACCTGTTCATATCGGTGACGGCGATTCTCAC ATTTGGAACGAGACC
88	GGTCTCA	CTGA	TTTAT TCAGG GCACT ACCCG GAGCT	AGTGGTA GGTAGTT TTGCCCA GCCC	ATCGGTGAC GGCGATTCT CACATTT	GGAA	CGAGACC	97	4	4_16	GGTCTCACTGATTTATTCAGGGCACTACCCGGAGCTAGTGG TAGGTAGTTTTGCCCAGCCCATCGGTGACGGCGATTCTCAC ATTTGGAACGAGACC
89	GGTCTCA	CTGA	TTTAT TCAGG GCACT ACCCG GAGCT	TTGGGCC GATAAGT TGAGTAC CCTG	ATCGGTGAC GGCGATTCT CACATTT	GGAA	CGAGACC	97	4	4_17	GGTCTCACTGATTTATTCAGGGCACTACCCGGAGCTTTGGG CCGATAAGTTGAGTACCCTGATCGGTGACGGCGATTCTCAC ATTTGGAACGAGACC

90	GGTCTCA	CTGA	TTTATTCAGGGCACTACCCGGAGCT	GCATAGGGTTCCACGCCAGTGTATG	ATCGGTGACGGCGATTCTCACATTT	GGAA	CGAGACC	97	4	4_18	GGTCTCACTGATTTATTCAGGGCACTACCCGGAGCTGCATAGGGTTCCACGCCAGTGTATGATCGGTGACGGCGATTCTCACATTTGGAACGAGACC
91	GGTCTCA	CTGA	TTTATTCAGGGCACTACCCGGAGCT	ATTCAGGGTTCCCACATCACGCAAA	ATCGGTGACGGCGATTCTCACATTT	GGAA	CGAGACC	97	4	4_19	GGTCTCACTGATTTATTCAGGGCACTACCCGGAGCTATTCAGGGTTCCCACATCACGCAAAATCGGTGACGGCGATTCTCACATTTGGAACGAGACC
92	GGTCTCA	CTGA	TTTATTCAGGGCACTACCCGGAGCT	GCATTCTGGTTCGCAGCTATATCGG	ATCGGTGACGGCGATTCTCACATTT	GGAA	CGAGACC	97	4	4_20	GGTCTCACTGATTTATTCAGGGCACTACCCGGAGCTGCATTCTGGTTCGCAGCTATATCGGATCGGTGACGGCGATTCTCACATTTGGAACGAGACC
93	GGTCTCA	CTGA	TTTATTCAGGGCACTACCCGGAGCT	GCATAACACAAGTACGGCTACGCAG	ATCGGTGACGGCGATTCTCACATTT	GGAA	CGAGACC	97	4	4_21	GGTCTCACTGATTTATTCAGGGCACTACCCGGAGCTGCATAACACAAGTACGGCTACGCAGATCGGTGACGGCGATTCTCACATTTGGAACGAGACC
94	GGTCTCA	CTGA	TTTATTCAGGGCACTACCCGGAGCT	GAAGGAGGCACATCCTTAAACCCGT	ATCGGTGACGGCGATTCTCACATTT	GGAA	CGAGACC	97	4	4_22	GGTCTCACTGATTTATTCAGGGCACTACCCGGAGCTGAAGGAGGCACATCCTTAAACCCGTATCGGTGACGGCGATTCTCACATTTGGAACGAGACC
95	GGTCTCA	CTGA	TTTATTCAGGGCACTACCCGGAGCT	GGCTATGTCCGTAACACTCCTAGCA	ATCGGTGACGGCGATTCTCACATTT	GGAA	CGAGACC	97	4	4_23	GGTCTCACTGATTTATTCAGGGCACTACCCGGAGCTGGCTATGTCCGTAACACTCCTAGCAATCGGTGACGGCGATTCTCACATTTGGAACGAGACC
96	GGTCTCA	CTGA	TTTATTCAGGGCACTACCCGGAGCT	GAGCGACAATCGACTTTCGTGGATC	ATCGGTGACGGCGATTCTCACATTT	GGAA	CGAGACC	97	4	4_24	GGTCTCACTGATTTATTCAGGGCACTACCCGGAGCTGAGCGACAATCGACTTTCGTGGATCATCGGTGACGGCGATTCTCACATTTGGAACGAGACC

Supplementary Table 4. Primers used for DMX

	**Sequence (5' -> 3')**
Forward library primer	atactacCGTCTCgaggaGGTGGATCAGGAGGTTCG
Reverse library primer	gcattacCGTCTCcggaaCCACTTCCACCGCTTCC
DMX 1_rv	TCATGCCGCGACGCATACTTTAGAC
DMX 2_fw	CTGGTTGGGATCAGTCGCTTAGTGC
DMX 3_rv	CTGGGTACTGTCCCAAGGACCACTC
DMX 4_fw	CGACACCGAACGTGCGACAAAACTA
DMX 5_rv	CTCTGCGACCTAGCCGGTTTATCCA
DMX 6_fw	TTTATTCAGGGCACTACCCGGAGCT

Supplementary Table 5. Cost breakdown for DMX (green), and comparison to SAPP (blue) for the same number of designs.

	Library complexity			
**Synthetic DNA**	**100**	**500**	**1000**	**2000**
Twist oligo pool (250-300 nt, list prices)	$ 1,030.00	$ 2,060.00	$ 3,090.00	$ 4,121.00
subtotal:	$ 1,030.00	$ 2,060.00	$ 3,090.00	$ 4,121.00
**Cloning**				
BsaI	$ 76.26	$ 152.52	$ 305.05	$ 610.10
Salt T4 ligase	$ 115.20	$ 230.40	$ 460.80	$ 921.60
subtotal:	191.46	$ 382.92	$ 765.85	$ 1,531.70
**Sequencing**				
Minion flow cell (ONT)	$ 600.00	$ 600.00	$ 600.00	$ 600.00
Library Prep kit (ONT)	$ 95.00	$ 95.00	$ 95.00	$ 95.00
subtotal:	695.00	$ 695.00	$ 695.00	$ 695.00
**Plasticware**				
ECHO plates	$ 15.04	$ 30.07	$ 60.14	$ 120.29
1536w rxn plates	$ -	$ 50.93	$ 101.87	$ 203.73
245mm Square BioAssay Dishes	$ 20.31	$ 20.31	$ 40.63	$ 81.25
subtotal:	35.35	$ 101.32	$ 202.64	$ 405.27
**DMX Total Cost for the library ($)**	**1,951.81**	**3,239.24**	**4,753.49**	**6,752.97**
**DMX Cost / design ($)**	**19.52**	**6.48**	**4.75**	**3.38**
eBlocks Gene Fragments subtotal:	$ 2,100	$ 10,500	$ 21,000	$ 42,000
eBlocks Cloning subtotal:	$ 124	$ 622	$ 1,244	$ 2,488
**SAPP DNA Total cost ($)**	**2,224.40**	**11,122.00**	**22,244.00**	**44,488.00**
**SAPP DNA cost ($) / design**	**22.24**			
**Saving factor, DMX compared to eBlocks**	**1.14**	**3.43**	**4.68**	**6.59**

Supplementary Table 6. Reagents and materials for SAPP.

**Cloning**	**Supplier**	**Cat#**
BsaI-HF®v2	NEB	R3733L
T4 DNA Ligase	NEB	M0202L
Entry vector (e.g. LM0627, Midiprep)	ZymoResearch	D4201
BL21(DE3) Competent E. Coli	NEB	C2527I
NEB Stable for GGA vector propagation with *ccdb* lethal gene	NEB	C3040H
**Protein Expression**		
Terrific broth II	MP Biomedicals	113046032
Glycerol	MilliporeSigma	G5516-4L
Dextrose (D-Glucose), Anhydrous	FisherScientific	D16-1
D-Lactose monohydrate	MilliporeSigma	61345-1KG
Kanamycin sulfate	FisherScientific	AC450811000
**Protein Purification**		
B-PER™	ThermoFisher	78248
Benzonase® Nuclease	MilliporeSigma	E1014-25KU
PMSF	MilliporeSigma	11359061001
Lysozyme from chicken egg white	MilliporeSigma	L6876
HisPur™ Ni-NTA Resin	ThermoFisher	88223
Cytiva S200/S75 Increase 5-150	Cytiva	29148722
**Buffers**		
Trizma® base	MilliporeSigma	T1503-10KG
NaCl	FisherScientific	BP358-212
Imidazole	MilliporeSigma	1370981000
**Plasticware**		
Tips	Opentrons	999-00013

Armadillo PCR plate	FisherScientific	AB2396
96-well 2 mL Deepwell culture plate	FisherScientific	278752
Breathable membrane (Diversified Biotech Breathe Easier) **Do not use the "Breathe Easy" from the same manufacturer, we had poor expression results with the "Breath Easy" film.**	MilliporeSigma	Z763624
96-well fritted plate 25 µm	Agilent	200967-100
96-well filter plate 0.2 µm	Agilent	203940-100
96-well receiver plate	ThermoFisher	249944
384-well HPLC collection plate	Greiner Bio-One	781270

Supplementary Table 7. SAPP Buffers.

Name	Recipe	amount	comment
**TB-2 Media**	from MP Biomedicals	50 g/L	prepare in 1L flasks, autoclave
**5052 (50x stock)**	Glycerol	250 g/L	sterile filter
	D-(+)-Glucose monohydrate	27.5 g/L	
	α-Lactose monohydrate	100 g/L	
**Autoinduction media (1 L)**	50x 5052, sterile	20 mL	prepare sterile (under flame)
	1 M $MgSO_4$, sterile	2 mL	
	1000 x antibiotic, sterile	1 mL	e.g. Kanamycin final 50 µg/mL
	TB2	to 1 L	
**HIS WASH BUFFER**	Tris	20 mM	pH 8
	NaCl	300 mM	
	Imidazole	25 mM	
**HIS ELUTION BUFFER**	Tris	20 mM	pH 8
	NaCl	300 mM	
	Imidazole	500 mM	
**His resin stripping buffer**	NaOH	500 mM	
	EDTA-2Na·$2H_2O$	50 mM	
**SNAC cleavage buffer**	CHES	100 mM	pH 8.6
	Acetone Oxime	100 mM	
	NaCl	100 mM	
	Guanidinium HCl	500 mM	

Supplementary Table 8. Reagents and materials for DMX.

**Cloning**	**Supplier**	**Cat#**
KAPA HiFi HotStart ReadyMix	Roche	KK2602
EvaGreen Dye	Biotium	31000
Zymoclean Gel DNA Recovery Kits	Zymo Research	D4007/D4008
BsmBI-v2 NEBridge Golden Gate Assembly kit	NEB	E1602S
DNA Clean & Concentrator-5	Zymo Research	D4003
E. Cloni EXPRESS BL21(DE3) electrocompetent cells	Lucigen	60300-1
BsaI-HF®v2	NEB	R3733L
Salt-T4 DNA Ligase	NEB	M0467L
Zyppy Plasmid Miniprep	Zymo Research	D4036
dsDNA Quantitation, High Sensitivity	Invitrogen	Q32851
**Plasticware**		
Breathe Easier Plate Seal	Fisher Scientific	NC1664397
Axygen® Foil Plate Seal	Fisher Scientific	PCR-AS-600
ECHO-qualified 384-well plates	Beckman Coulter	C74290
ECHO-qualified polypropylene 1536-well plate	Greiner	782270
Bioassay plates	Corning	431111

Supplementary Table 9. CryoEM data collection, refinement, and validation statistics

	RSV-F<br>SC-DM/cb13<br>PDB:9YCF<br>EMDB-72769
Data collection and processing	
Magnification	36,000 ×
Voltage (kV)	200
Electron exposure (e–/Å^2)	50
Defocus range (μm)	1-2
Pixel size (Å)	0.4425
Symmetry imposed	C1
Initial particle images (no.)	866,428
Final particle images (no.)	89,296
Map resolution (Å)	4.62
FSC threshold	0.143
Map resolution range (Å)	4.0-6.0
Refinement	
Initial model used (PDB code)	
Model resolution (Å)	4.62
FSC threshold	0.143
Model resolution range (Å)	4.0-6.0
Map sharpening *B* factor (Å^2)	217.3
Model composition	
Non-hydrogen atoms	7045
Protein residues	1423
Ligands	N/A
B factors (Å^2)	
Protein	178.59
Ligand	N/A
R.m.s. deviations	
Bond lengths (Å)	0.002
Bond angles (°)	0.525
Validation	
MolProbity score	1.39
Clashscore	2.18
Poor rotamers (%)	0.0
Ramachandran plot	
Favored (%)	94.11
Allowed (%)	5.89
Disallowed (%)	0
