# Supplementary material for: Accelerating protein design by scaling experimental characterization
Source: Nat Commun. 2026 Aug 20;17:9945. doi: 10.1038/s41467-026-76740-9 (PMC13586242; doi:10.1038/s41467-026-76740-9)
Supplement: Supplementary file 3 [file 41467_2026_76740_MOESM3_ESM.pdf]

nature portfolio

Peer Review File

Accelerating protein design by scaling experimental characterization

Corresponding Author: Dr David Baker

This file contains all reviewer reports in order by version, followed by all author rebuttals in order by version.

Version 0:

Reviewer comments:

Reviewer #1

(Remarks to the Author)
This manuscript seeks to tackle a key bottleneck in protein science. How to inexpensively and rapidly access pure proteins quickly in a generalized way. This is especially a challenge in protein design, where computational designs on a computer happen significantly faster than experimentation in the lab.

Here, the authors describe the development of a semi-automated workflow for protein production and characterization (SAPP). A key feature is the DNA demultiplexing protocol (DMX) designed to lower per-design costs in large protein design campaigns. The streamlined SEC-based pipeline is a particular strength, as it provides data-rich readouts (e.g., yield, oligomeric state, dispersity) for every variant and clearly differentiates this platform from many existing approaches, as illustrated by the RSV binder oligomerization case study. This work provides a practical framework that can, in principle, be adopted by many other laboratories, as long as they have the robotic facilities. The work is important because it was shown to bridge the gap between computational design and experimental validation at scale.

While the work is technically sound, addresses a need in the protein design field, and will likely be of broad practical value to groups seeking to scale experimental testing (if it can be reproduced by others), there are some clarifications needed. In addition, more explicit discussion of the limitations and scope are required. Finally, it would be great to provide a clear "how-to" guide on how others would exactly replicate this in their laboratories. I think this is critically needed.

Concerns.
1. The authors assess polyclonality of SAPP by reporting sequencing results for 929 Golden Gate Assembly (GGA) reactions with insert size below 314bp. Because cloning and assembly efficiency can depend on sequence composition and properties, additional explanation of how these 929 designs were selected and whether there are identifiable sequence- or length-dependent conditions where performance deteriorates (e.g. decreased clonal purity for longer inserts) would help assess how generalizable the reported clonal purity is across different protein sequence contexts.
2. The authors need to add concrete design guidelines that users should consider maintaining robust performance of this workflow and minimize fitness-driven false negatives in protein expression.
3. The SAPP pipeline is described as producing SEC purified, concentration normalized protein samples consolidated into 96 well plates, which suggests that these materials can be used directly in downstream functional or activity based assays. For the fluorescent protein and RSV case studies, it appears that SEC data are used to select promising variants and that a subset is then subjected to biophysical or neutralization assays, however, it is not clearly stated whether these assays were performed directly using the SEC purified, plate consolidated material generated by SAPP, or whether additional re purification or concentration steps were involved. This needs to be added.
4. If re-purification was needed, please provide a new alternative example in which functional readouts are obtained directly from SAPP outputs. This would elevate the impact of the paper.
5. The SAPP workflow, as described, is naturally optimized for in vivo expression in E. coli using autoinduction media, followed by Ni NTA affinity purification and SEC. This is an excellent fit for many designed mini binders, small enzymes, and symmetric assemblies that express well as soluble proteins in bacteria, and the examples in the manuscript fall into this class. However, this also means that the current implementation is less directly applicable to other important target categories, such as membrane proteins, secreted or glycosylated proteins, and designs that inherently require specific folding or post translational modifications. It would be very helpful if the authors could expand the discussion to more explicitly define the scope of SAPP, what kinds of targets it is best suited for, where it has been successfully applied within

their institute, and where they anticipate significant limitations.
6. What is the largest monomer protein made? Can this make 50? 75? 100? kDa proteins? Where do limits of DNA synthesis occur versus SAPP?
7. How does DMA compare or contract to other previously described multiplexed DNA pool based construction? Such as the work of Phil Romero or Sean Lund (https://pubs.acs.org/doi/10.1021/acssynbio.3c00694)
8. The authors should add an appendix that provide the exact how to guide on replicating all workflows. I apologize if this was missed.

(Remarks on code availability)

Reviewer #2

(Remarks to the Author)
The manuscript by Qian et al describes a protocol for rapid screening of de novo designed proteins.
ML-based protein design recently demonstrated exciting potentials for all areas of life sciences. Due to the availability and user-friendly computational platform, this tool is becoming accessible to an increasing number of researchers for diverse applications.
This manuscript aims to present a protocol for rapid experimental screening of a large number of designs as implemented in the Baker lab. This protocol could therefore be useful for other researchers, although hopefully, the success rate will improve to eliminate the need to test hundreds or thousands of designs.
The manuscript, however, does not present any new technology. The main difference to the existing protocols is streamlining of the process and an inclusion of SEC purification, which is particularly suited for the analysis of oligomerization state.
The protocol is demonstrated on two examples, which to some degree resemble previously published optimizations from the same lab; first for the redesigned FPs, which is similar to the redesign of a TEVp (Sumida et al., JACS 2024). As this is intended as a demonstration of a protocol, few details are provided. Nevertheless, it would be of interest to explain why some designs have substantially larger elution volumes (Fig 2de) than the monomer. Also, the large Stokes shift in Fig 2e is of interest, but again, no explanation on the nature of mutations is provided, unexpected since the residues close to the chromophore have not been modified.
The second demonstration is on oligomerized RSV binders, where 116 designs have been tested, which could in principle also be done without resorting to the large-scale protocol. This part of the study resembles the effect of oligomerizers of SARS-CoV-2 binders (Hunt et al., SciTranslMed 2022) or a trimer against hemagglutinin by the same group (Strauch et al., NatBiotech 2017). In this particular case, interestingly, larger oligomers do not incur any advantage in comparison to a dimer. Structure of a monomeric binder with RSV F (SC-DM) is provided but not with an oligomeric, much stronger binder, which is the desired result.

For both demonstrations sequences of binders should be included in the supplement, along with their characterized properties.

The DMX pipeline, on the other hand, is not demonstrated on a particular example. The authors claim that this strategy is more cost-effective for >400 constructs, although it requires a substantial amount of work, for coding and decoding sequences, while the functional characterization of each variant remains to be done in the same manner as for other designs (SEC seems difficult to perform for 2000 variants ?).
Other strategies, such as e.g. ribosomal or RNA-display, or encapsulation based strategies might be better suited to match each sequence for a protein phenotype in much more effective ways than this protocol. In that type of assay, only the selected variants need to be sequenced.

(Remarks on code availability)

Version 1:

Reviewer comments:

Reviewer #1

(Remarks to the Author)
The authors have addressed my concerns. I believe others will find it useful.

(Remarks on code availability)

Reviewer #2

(Remarks to the Author)
Manuscript provides the protocol/workflow for semi-automated high throughput (designed) protein generation and characterization that could be useful for other labs that desire to test large number of protein variants. Authors have added

some missing information and provided appropriate clarification to the issues raised by the reviewers.

(Remarks on code availability)

Open Access This Peer Review File is licensed under a Creative Commons Attribution 4.0 International License, which permits use, sharing, adaptation, distribution and reproduction in any medium or format, as long as you give appropriate credit to the original author(s) and the source, provide a link to the Creative Commons license, and indicate if changes were made.
In cases where reviewers are anonymous, credit should be given to 'Anonymous Referee' and the source.
The images or other third party material in this Peer Review File are included in the article's Creative Commons license, unless indicated otherwise in a credit line to the material. If material is not included in the article's Creative Commons license and your intended use is not permitted by statutory regulation or exceeds the permitted use, you will need to obtain permission directly from the copyright holder.
To view a copy of this license, visit https://creativecommons.org/licenses/by/4.0/

Reviewer #1 (Remarks to the Author):

This manuscript seeks to tackle a key bottleneck in protein science. How to inexpensively and rapidly access pure proteins quickly in a generalized way. This is especially a challenge in protein design, where computational designs on a computer happen significantly faster than experimentation in the lab.

Here, the authors describe the development of a semi-automated workflow for protein production and characterization (SAPP). A key feature is the DNA demultiplexing protocol (DMX) designed to lower per-design costs in large protein design campaigns. The streamlined SEC-based pipeline is a particular strength, as it provides data-rich readouts (e.g., yield, oligomeric state, dispersity) for every variant and clearly differentiates this platform from many existing approaches, as illustrated by the RSV binder oligomerization case study. This work provides a practical framework that can, in principle, be adopted by many other laboratories, as long as they have the robotic facilities. The work is important because it was shown to bridge the gap between computational design and experimental validation at scale.

We thank the reviewer for their encouraging comments. We wanted to point out that while indeed many of the steps can be simplified with the use of robotics, we are aware that not every lab may have access to such infrastructure. In an attempt to ensure that as many people as possible could adopt our protocols, we also describe in detail (in SI) how to perform the SAPP protocol without robots. Specifically, high-throughput cloning without the need for an ECHO, and how to re-purpose an AKTA for high-throughput SEC in the absence of an HPLC. Fraction pooling is intended to be performed with an OT-2, an affordable open-source liquid handler, although we have also done these steps by hand in the past with only minimal additional time, further showcasing how our protocols are meant to democratize high-throughput protein testing.

While the work is technically sound, addresses a need in the protein design field, and will likely be of broad practical value to groups seeking to scale experimental testing (if it can be reproduced by others), there are some clarifications needed. In addition, more explicit discussion of the limitations and scope are required. Finally, it would be great to provide a clear “how-to” guide on how others would exactly replicate this in their laboratories. I think this is critically needed.

We fully agree with this reviewer that one of the key added value of our manuscript is ensuring that what we describe can readily be adopted and deployed in labs around the world. For the explicit purpose, we included a very detailed step-by-step guide in the supplementary information of the paper, for both SAPP and DMX. These supplementary methods go into much more detail than a “typical” methods section, and were written on the basis of our internal SOP. These sections contain

detailed protocol information, materials, and handling procedures based on our experience teaching this protocol to dozens of researchers (rotation students, masters students, visiting scholars, PhD students, postdoc, research assistants) over 2 years. We hope that its content satisfies the reviewer, and we welcome additional comment/suggestion if it is felt that the current information provided is lacking for a full reproduction.

Regarding explicit discussion of the limitation and scope, the main aspect concerning SAPP is the risk associated with polyclonality, which makes the protocol well-suited for screening campaigns where some potential false negatives are acceptable, but not necessarily in cases where absolute clonal certainty is required. This is also why we developed DMX, and decided to publish these two methods together since we see them as complementary. We mention the potential issue of polyclonality in the results section: "*While highly efficient, our protocol may still occasionally yield false negative results, and thus is primarily intended for screening purposes.*" We have now also included an explicit point in the discussion about this limitation (page 9): "We note that the small levels of polyclonality resulting from synthesis error are length-dependent, which can complicate using the protocol for longer proteins."

We have also included an explicit discussion regarding the (potential) limitation regarding application of this protocol to non-designed proteins. While we have explicitly developed this protocol for wetlab validation of designed proteins, we realize that we did not explicitly state that in the discussion. We have now included a section in order to make that point explicit (page 9): "*This protocol was developed and optimized for wetlab validation of high-yielding proteins such as engineered and designed sequences, and may struggle with proteins that require complex expression and purification protocols*."

In our experience, these two points constitute the main limitations of SAPP, and we have now made these elements more explicit in the manuscript.

Concerns.

1. The authors assess polyclonality of SAPP by reporting sequencing results for 929 Golden Gate Assembly (GGA) reactions with insert size below 314bp. Because cloning and assembly efficiency can depend on sequence composition and properties, additional explanation of how these 929 designs were selected and whether there are identifiable sequence- or length-dependent conditions where performance deteriorates (e.g. decreased clonal purity for longer inserts) would help assess how generalizable the reported clonal purity is across different protein sequence contexts.

The 929 designs used for the NGS analysis were a randomized selection of glycerol stocks from different users, which are representative of our use-cases. The

sequencing length limit was imposed by the sequencing experiment performed, which was 150 bp, paired-end. Because 300 bp has constituted the vast majority of designs tested at our institute during the time-frame between the establishment of this assay and the submission of this manuscript, we deemed that it was representative of the bulk of our experimental workflows. We did not identify any sequence features that would explain deterioration, which might in part be due to the DNA sequence optimization used as part of the workflow (described in SI, and code made available in the github repository), in particular the suppression of k-mers to avoid synthesis issues.

We do agree with the reviewer that clonal purity is likely to be length-dependent, especially since virtually all "clonal impurities" appear to be from synthesis errors, and those are bound by the manufacturer's error rate, which is a function of length. While we do not have sequencing results in the longer length regime, our LC-MS results on longer proteins suggest that it is not a significant problem since even for proteins up to 50 kDa (450 aa, 1350 bp) LC-MS did not reveal major cases of contamination.

We have now also included an explicit statement in the discussion regarding the length-dependence of synthesis accuracy to make the reader aware. This discussion point was added alongside the discussion about the limitation of polyclonality (cf. answer above) (page 9): "We note that the small levels of polyclonality resulting from synthesis error are length-dependent, which can complicate using the protocol for longer proteins*."*

Importantly, our LC-MS data does not point to this being an issue, but we thank the reviewer for his question, and hope that our explicit mention of this potential limitation will be informative to the reader.

2. The authors need to add concrete design guidelines that users should consider maintaining robust performance of this workflow and minimize fitness-driven false negatives in protein expression.

Our goal was to establish a workflow that was agnostic to the input sequence, enabling high-throughput testing of any designed protein. This protocol has been used internally across hundreds of design campaigns, each with their own functional objectives, ranging for fluorescent proteins and RSV binders (this manuscript), binders against dozens of other targets, enzymes and nanomaterial components to mention a few. For each, no specific "expression" guidelines were applied when selecting sequences for testing. In our experience, designed proteins in general are well-suited for expression/purification with this protocol. Our experience mostly relates to ProteinMPNN-designed sequences, although other

approaches may also be suitable such as ESM-based sequence design (cf. https://www.biorxiv.org/content/10.1101/2022.12.21.521521v1)

While we do not have specific design guidelines, and feel that this would partially defeat the purpose of this protocol (which is intended to be design-agnostic), we have now explicitly stated in the discussion that the protocol was established and validated on engineered and designed proteins (cf. see above) (page 9): "*The protocol was developed and optimized for wetlab validation of high-yielding proteins such as engineered and designed sequences, and may be less well suited for proteins that require complex expression and purification protocols*"

3. The SAPP pipeline is described as producing SEC purified, concentration normalized protein samples consolidated into 96 well plates, which suggests that these materials can be used directly in downstream functional or activity based assays. For the fluorescent protein and RSV case studies, it appears that SEC data are used to select promising variants and that a subset is then subjected to biophysical or neutralization assays, however, it is not clearly stated whether these assays were performed directly using the SEC purified, plate consolidated material generated by SAPP, or whether additional re purification or concentration steps were involved. This needs to be added.

We thank the reviewer for asking for clarification. For the fluorescent protein studies and RSV F protein binding assays, the material was directly from the SEC purified samples using the SAPP pipeline.
For the neutralization studies, all initial material was produced by SAPP (4 mL autoinduction culture grown in 96 well deep well plates).
Due to the lag between initial binding screening and neutralization studies, proteins had to be-expressed. The subset re-expressed for neutralization studies were also obtained from SAPP. Now more explicitly state (page 21):
"The next day, serial 3-fold dilutions in culture media were performed on minibinder samples obtained directly with SAPP"
In the case of the fluorescent proteins, yields from the first SAPP purification of a 4 mL autoinduction culture grown in 96 well deep well plates was around 0.2 - 0.25 mg (highlighted below, also included in Supplementary Figure 1), corresponding to a liter equivalent yield between 50 - 60 mg (shown below). All data shown were collected on the pooled samples of this SAPP run (all higher than 20 µM, if they could be purified solubly). No further concentration was needed and absorbance and fluorescence measurements for all data shown could be carried out on the pooled and diluted sections directly at 20 µM.
This is now explicitly stated in the methods section (page 17):
"*Experiments: Following SAPP purification of 88 designs and 12 respective WT controls, pooled fractions containing soluble fluorescent proteins from SEC in PBS*

were tested for their fluorescence properties in a plate reader (Agilent BioTek Neo2) at a concentration of 20 µM.”

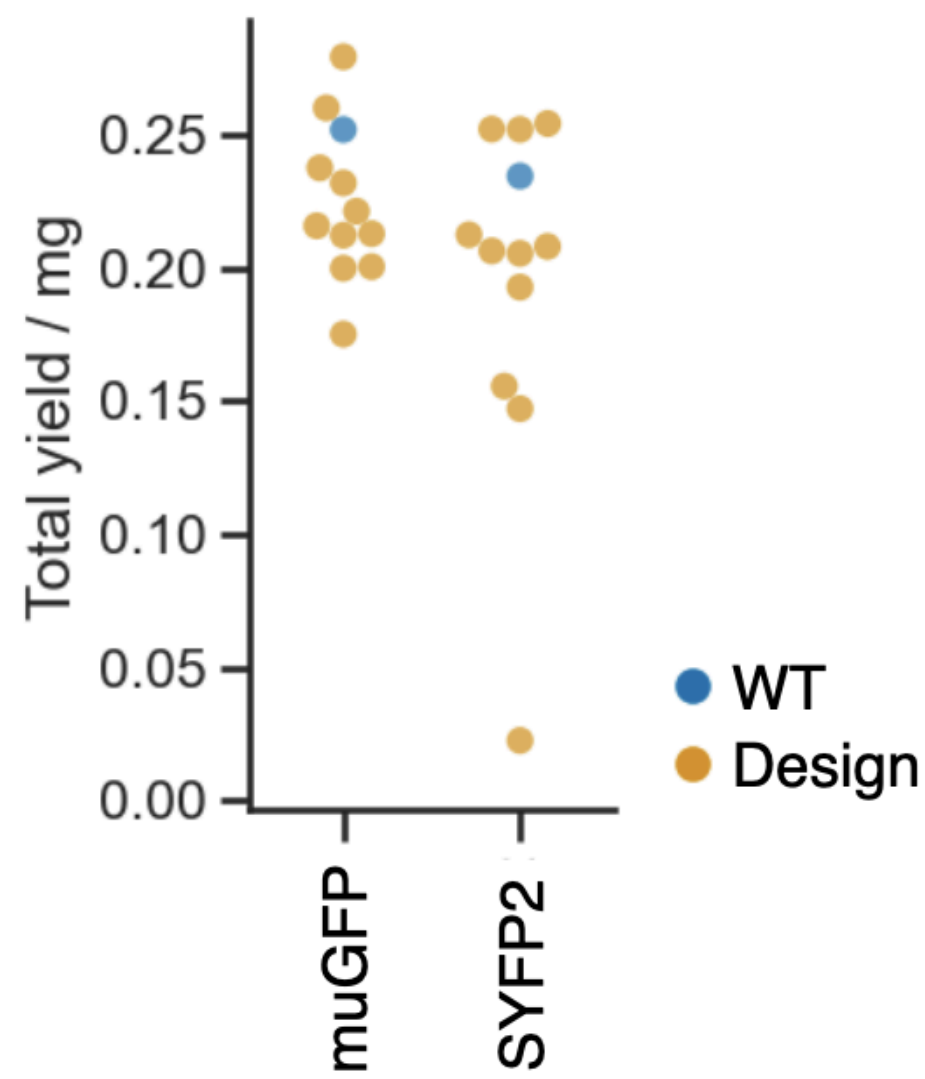

4. If re-purification was needed, please provide a new alternative example in which functional readouts are obtained directly from SAPP outputs. This would elevate the impact of the paper.

As mentioned above the binding assays and fluorescent protein experiments were done using SAPP-produced material, as well as the neutralization studies. We also note that SAPP was recently used for the production of 384 isotopically-labelled proteins for direct use in NMR, without concentration
(cf. https://www.biorxiv.org/content/10.64898/2026.02.16.706194v1)

5. The SAPP workflow, as described, is naturally optimized for in vivo expression in E. coli using autoinduction media, followed by Ni NTA affinity purification and SEC. This is an excellent fit for many designed mini binders, small enzymes, and symmetric assemblies that express well as soluble proteins in bacteria, and the examples in the manuscript fall into this class. However, this also means that the current implementation is less directly applicable to other important target categories, such as membrane proteins, secreted or glycosylated proteins, and designs that inherently require specific folding or post translational modifications. It would be very helpful if the authors could expand the discussion to more explicitly define the scope of SAPP, what kinds of targets it is best suited for, where it has been successfully applied within their institute, and where they anticipate significant limitations.

The reviewer makes an excellent point, and we fully agree that indeed the protocol is well-suited for (small) designed proteins, which constitute the bulk of test-cases. While we have not tested its performance on natural proteins, membrane proteins, or cases where complex post-translational modifications are required, we anticipate that yields are going to be lower for such cases. Secretion from mammalian cells (e.g. Expi293) in 96w format instead of E. coli could alleviate some of the issues of complex targets, but we did not test this as it was outside of the scope of this work. The primary objective for the development of this protocol was the testing of designed proteins expressed from E. coli.

6. What is the largest monomer protein made? Can this make 50? 75? 100? kDa proteins? Where do limits of DNA synthesis occur versus SAPP?

Yes, larger constructs have been made, including current unpublished work from the authors that successfully produced single 60 kDa monomeric proteins. Fundamentally SAPP is limited only by the quality of the DNA supplied by the manufacturer, and the tolerance of the assay/readout to polyclonality as discussed earlier. Since this tradeoff is specific to the design objective, it is not possible to put a single hard ceiling number on it.

7. How does DMA compare or contract to other previously described multiplexed DNA pool based construction? Such as the work of Phil Romero or Sean Lund (https://pubs.acs.org/doi/10.1021/acssynbio.3c00694)

We thank the reviewer for the question. All three papers tackle similar bottlenecks (i.e. the cost and scalability of DNA synthesis for protein engineering), but they each address a different piece of the pipeline and end with different deliverables.

Key differences:
All three papers use Golden Gate Assembly (GGA) with BsaI overhangs, but the format differs. DAD from Lund et al., assembles one single gene per reaction using barcode-indexed PCR from the pool, then transforms and picks 4 colonies for sequencing. OMEGA from Freschlin et al., assembles multiple genes per reaction in subpools indexed by orthogonal primer pairs, producing a pooled library without colony picking. Our DMX protocol takes a different approach: a library of genes are cloned into a dual-purpose vector, transformed, colonies are picked and barcoded 384-well plates, then sequence-verified using long-read sequencing. **The key distinction is that DMX focuses on the production of arrayed clonal and sequence-verified cultures instead of gene assembly, which can then feed directly into the SAPP protein production pipeline.**

These papers are more complementary than competitive. Lund et al., establish the proof-of-principle for gene assembly from oligo pools. OMEGA extends this method into multiplexed pooled assembly optimized for library-scale functional screening.

Our DMX method established multiplexed barcoding for sequencing to deliver arrayed clonal and sequence-verified constructs. We explicitly cite both Lund et al., and OMEGA (references 49 and 50, respectively) as related approaches to multiplexed GGA of long genes of interest. The papers satisfy different needs: OMEGA is well suited for the generation of pooled libraries of large assembled genes from oligo pools. Our DMX method is appropriate for large-scale barcoding and sequencing of pooled libraries for generating arrayed clonal constructs, irrespective of the original pool modality (e.g. DropSynth, OMEGA, etc..).

8. The authors should add an appendix that provide the exact how to guide on replicating all workflows. I apologize if this was missed.

We included detailed step-by-step guides in the supplementary methods, and share all necessary code on github https://github.com/bwicky/SAPP_DMX. The supplementary methods are much more detailed than a “standard” method section, and represent our internal SOP used at the institute, including detailed handling steps. We hope that its content is in-line with the expectation of the reviewer, and we are confident that will help other people adopt our protocols.

Reviewer #2 (Remarks to the Author):

The manuscript by Qian et al describes a protocol for rapid screening of de novo designed proteins.
ML-based protein design recently demonstrated exciting potentials for all areas of life sciences. Due to the availability and user-friendly computational platform, this tool is becoming accessible to an increasing number of researchers for diverse applications.
This manuscript aims to present a protocol for rapid experimental screening of a large number of designs as implemented in the Baker lab. This protocol could therefore be useful for other researchers, although hopefully, the success rate will improve to eliminate the need to test hundreds or thousands of designs.
The manuscript, however, does not present any new technology. The main difference to the existing protocols is streamlining of the process and an inclusion of SEC purification, which is particularly suited for the analysis of oligomerization state.

We thank the reviewer for their comments on our manuscript, and thank the editor for the opportunity to address the points raised by the reviewer. We agree with the reviewer that aspects of SAPP are indeed streamlining technologies that may already be conducted in isolation, however we make the point that it is the end-to-end orchestration of these parts that makes SAPP distinctively efficient and enabling. We would like to point out that this goes well beyond simply combining known protocols; important optimizations at the molecular biology level were necessary to achieve these results, and we also provide a robust code-base (provided with a permissive license on github) to enable the execution of these workflows in a systematic and automated way, greatly accelerating wetlab execution, which we do not consider trivial, and required significant development and optimization.

In addition to the aspects that may be considered streamlining, we also developed and validated a high-throughput cloning protocol that significantly simplifies end-to-end execution, and actually makes this process possible at scale. This also involved the design of a suitable vector library to ensure cross-compatibility which we make available. To the best of our knowledge, nothing of the sort has been reported before, and this is further encouraged by the fact that we have often received very positive feedback and surprise of the scale at which we could perform this protocol when presenting these results.

Perhaps the reviewer was only referring to SAPP in their comment, but we also wanted to point out that DMX represents multiple innovation of its own, including; isothermal DNA barcoding directly in bacterial lysate by GGA, combinatorial barcoding, and demultiplexing by ONT sequencing. None of these innovations, on their own or combined have been reported to the best of our knowledge.

So while we agree with this reviewer that some aspects may appear as streamlining, we would argue that as a whole, the two methods that we report are much more than this, include molecular biology, robotic and software innovation, and that the sum of its parts is much more than its individual components, which is where we think this work is particularly valuable, and we hope enabling for the protein engineering and design community.

The protocol is demonstrated on two examples, which to some degree resemble previously published optimizations from the same lab; first for the redesigned FPs, which is similar to the redesign of a TEVp (Sumida et al., JACS 2024). As this is intended as a demonstration of a protocol, few details are provided. Nevertheless, it would be of interest to explain why some designs have substantially larger elution volumes (Fig 2de) than the monomer.

Designs that have a shifted main peak elution volume likely are aggregated homooligomers, detailed per-sample chromatogram of both the pre- and post 95C heating step can be seen in the Supplementary Figure 2, which is now explicitly mentioned in the Figure 2 caption: "*The top plots show elution profiles post-purification and after a 1-hour incubation at 95°C for all variants of that parental sequence (detailed chromatograms in Supplementary Figure 2)*". Some of the peaks in the chromatogram that are shifted to higher molecular weight for example the WT muGFP after the heating step retain the absorbance signal of the chromophore, indicating that the proteins aggregate, but remain - at least in part - active.

Also, the large Stokes shift in Fig 2e is of interest, but again, no explanation on the nature of mutations is provided, unexpected since the residues close to the chromophore have not been modified.

Design 4468 derived from SYFP2 in Figure 2e shows a longer Stokes shift (Ex/Em of 422/528 nm) compared to the WT (Ex/Em of 516/530 nm), the sequence alignment shows 82% identity.

```
4468      MVSPGAALFEGVVPVLVELDGDVNGHKFSVRGEGEGDAATGTLTLKLVNTTGKLPVPWPT  60
WT        MVSKGEELFTGVVPILVELDGDVNGHKFSVSGEGEGDATYGKLTLKLICTTGKLPVPWPT  60
          *** *  ** ****:*************** *******: *.*****: ***********

4468      LVTTLGYGVQAFARYPEHMRQHDFFKSAMPEGYKQERTIKFKDDGNYKTTAEVKFEGDTL  120
WT        LVTTLGYGVQCFARYPDHMKQHDFFKSAMPEGYVQERTIFFKDDGNYKTRAEVKFEGDTL  120
          **********.*****:**:************* ***** ********* **********

4468      VNRIELKGSDFKADGNILGHKLEYSYNDHNVYITADKANNGIKAKFTIEHDLKDGGKQLA  180
WT        VNRIELKGIDFKEDGNILGHKLEYNYNSHNVYITADKQKNGIKANFKIRHNIEDGGVQLA  180
          ******** *** ***********.**.********* :*****:*.*.*:::*** ***

4468      DHDQKNTPIGDGPVLLPEPHYLKYKITLSKDPNEKRDHMVLKETVTAAGIEPGMDELYK   239
WT        DHYQQNTPIGDGPVLLPDNHYLSYQSKLSKDPNEKRDHMVLLEFVTAAGITLGMDELYK   239
          ** *:************: ***.*: .************** * ******  *******
```

The closest Ex/Em FP to design 4468 in fp base is supernovagreen, which shows different spectra (wider Ex/Em peaks) and has only 27% sequence identity to design 4468.

A long Stokes shift GFP (LSSM GFP) is 77% identical to design 4468 and has similar Ex/Em values (400 / 510 nm), however it is not clear which mutations induce the long Stokes shift, mutations associated with causing the long Stokes shift from LSSM GFPs template (hyperfolder YFP) are not all in our design.

We provide the sequences in the supplementary data, however, we cannot clearly identify mutations associated with the spectral changes - highlighting the novelty of these mutations, and likely the importance of changing many sites at once to achieve these changed spectra.
Overall, these results highlight that native redesign with ProteinMPNN can introduce non-trivial sets of multi-point mutations that push the FPs into a sequence space with new and unexpected properties.

We have added these points more explicitly in the main text to the section on redesign of FPs (page 5): "*Overall, these results highlight that native redesign with ProteinMPNN can introduce non-trivial sets of mutations that push the FPs into a sequence space with altered spectral properties. Identification of these functional sequence variants would have been challenging without the combination of protein design methods and high throughput screening.*"

The second demonstration is on oligomerized RSV binders, where 116 designs have been tested, which could in principle also be done without resorting to the large-scale protocol. This part of the study resembles the effect of oligomerizers of SARS-CoV-2 binders (Hunt et al., SciTranslMed 2022) or a trimer against hemagglutinin by the same group (Strauch et al., NatBiotech 2017). In this particular case, interestingly, larger oligomers do not incur any advantage in

comparison to a dimer. Structure of a monomeric binder with RSV F (SC-DM) is provided but not with an oligomeric, much stronger binder, which is the desired result.

We thank the reviewer for this comment. The observation that the dimer performs as well as, or better than, higher-valency oligomers is intriguing. We hypothesize that this is due to the lateral orientation of the binding epitope on the RSV F protein, which contrasts with the apical epitopes found on SARS-CoV-2 RBD binders that block ACE2. In the latter case, a trivalent molecule can simultaneously engage three RBDs because the binding sites reside on a single horizontal plane.

For RSV, however, the side-oriented epitopes would require a binder to either wrap around the F protein in a ring-like configuration or cross-link distinct F proteins across the virion surface. As we did not conduct experiments to determine if our molecules facilitate inter-trimer cross-linking, we have omitted further discussion of that mechanism. Additionally, we did not pursue structural characterization of the oligomeric cb13 variants; all share the same minibinder domain, the binding mode of which we validated via cryo-EM.

For both demonstrations sequences of binders should be included in the supplement, along with their characterized properties.

We thank the reviewer for pointing out our omission. We have now added a table with all of the relevant binder and oligomer sequences in the supplementary information

Table S10. Sequences of RSV constructs. cb4 and cb13 monomers were expressed as N- or C-term fusions to each of the oligomers below linked by a GSG, with the addition of an N-terminal MSG motif and C-terminal SNAC and His tag, as described in the methods.

Molecule	Molecule type	Amino acid sequence
cb4	RSV minibinder	TLEKQLENLVRSAEVLVRHNDRLNAFFI LDQAKDLAERLNDPEKLRELKELEEEL
cb13	RSV minibinder	LEKQLENLVRTAEVLVRHNDRLNAFFIL EQAKDLAERLNDPETLREVKELHKEL
HALC1_004	Oligomer fusion	MIVSLEKHPGGVHIITLSSEENLENFVKE LKKLGAEVERLPEPNTVRVRAPEEVVEE ALKNTKFK
HALC1_008	Oligomer fusion	MLTPEELLERLRRHLEEEHGVVVPEERI LSVEATPTEVTLTWSRGDGRTGTARYTS DGRFEVEDP
HALC2_059	Oligomer fusion	MVKPITEEDVREAATAASPDYEVGEAKLI

		DEENNLWFVTLYKGDQKIYALIEDKNGE FTVHQIEL
HALC2_062	Oligomer fusion	MARVEYSYEKLNDTHYKLKLKVTYEYRK SPEARRLAEDLVQAFVDALSSLPFITVEY EVEEVEVE
HALC2_063	Oligomer fusion	MKVYEFPYPETGKKIIVIQGEKNIVIVVGN TAVVYYEGKWTYKENVTEEDIEKAKTEE GAKELAK
HALC2_064	Oligomer fusion	SKLKEQEELIDEISEKAKEFLLEIKEKYPG ELSEERYPGRVVLTYVNEEKGFSITVTIE LLNKEK
HALC2_065	Oligomer fusion	SEEEKPIVIDLNKTIERDGRKVKLVRATIT VDPETNTITIDIEYEGGPITKEDLLEAFKL AASKL
HALC2_067	Oligomer fusion	DKLVRVLSSSMIYYAERMTKGSTDPSDY DKALDDFYNYFLEQPFVDKETLEKAYEL ARKRLEELL
HALC2_068	Oligomer fusion	MIKVPEDLERIGRELRARGLDTKRLLEE GPKLYPELSIPDLMAIALYDHLNLDPEFL YRLLQQSR
HALC3_104	Oligomer fusion	KRIDEIESKLKHLEEFTTHLIKLMETMLEL LKLVSDGKSDSEEYKELLEKAEEYLKQA TEAAKKI
HALC3_109	Oligomer fusion	REEIEEAVKEAELKVLAIVLVALRSVSHY EPLSRLYESFLDALKKALSEEELKEVEKE AERIEKK
HALC3_110	Oligomer fusion	LEQILEELTELLERVDEIPLREALKRMLEL LVRVTQELKEVKDKVESLEKHLEELDKR VEEIEKK
HALC3_114	Oligomer fusion	VDEKEVKERFEEIESRLEELESKVREVE KKVEEVKKESDEKIDQLKTEFETKYNQI NNEINTLKN
HALC3_118	Oligomer fusion	MTRLEQLLAQGVDPFEVLREKIEKLKEI WKKYEEAKGEEKERYRDELLKLMMEVL ELMVELLSRR
HALC3_919	Oligomer fusion	SEELLEELRELLERLQELLELIEQGKITPE QLREAIALLIEVLQILYEALRELAEQLQRL REELG
SB175	Oligomer fusion	SEALEELEKALRELKKSTDELERSTEEL EKNPSEDALVENNRLIVENNKIIVEVLRII AKVLK
HALC4_135	Oligomer fusion	MEKFKEQLLEEVKKIVLETMTKVMEHLE

		KWFVTLAEIIITKSEEKLEELKETMEKSIE<br>ELRKEAE
HALC4_136	Oligomer fusion	MSPYKKAIEITKRLLELLLSNPELAKKNL<br>GGIATLISLLALISALDGTLDEKDIEPYIKK<br>LEESL
HALC4_140	Oligomer fusion	MEEVVLTSHNELHKKLDEVHDKIMSKLD<br>EIHEKLDEIISKLDEIESKLHEILNIVKEIKE<br>ILEKK
HALC5_169	Oligomer fusion	LLLEVMEKVFDEEQLKLIKEAAEREGNS<br>PVVISSIATLLLLERIEKIVKEIHDEVKKNN<br>EKQEKK
HALC5_176	Oligomer fusion	MDPKELEREALKNIIKLPKLIQDFKDSVM<br>KELNKIIELLEERRREIDEPLLPIIRKLQEE<br>LQKKE
C2-02802	Oligomer fusion	DLEERFERLYEKAKKLAEERGDERARR<br>MIELLRQLFETVGDPRILELLELLLQLLEG
C4-71-8	Oligomer fusion	PEEILERARESLERAREASERGDEEEFR<br>KAAEKALELAKRLVEQAKKEGDPWMVM<br>WAALVALWVALLALRNGDKEVFKKAAES<br>ALEVAKRLVEVASKEGDPEMVLLAAWVA<br>LFVAWLAWLFGDKEVFKKAAESALEVAK<br>RLVEVASKEGDPELVEEAAKVAEEVEKL<br>AEKQGDEEVREKAWETWMEVWLLWLE<br>VRLRKGGG
C4-81-4	Oligomer fusion	ELERESREAEKRLKEARLFAWAARLLGD<br>LKLLAKALIEEARAVQELARVACERGNR<br>DEAWDAFEKALEVFEEAVKVSEEAREQ<br>GDDEVLALALIAIALAVLALAEVACCLGIS<br>ELAELAWKMAEWVLEEARKVSEEAREQ<br>GDDEVLALALIAIALAVLALAEVACCRGN<br>KEEAERAYEDARRVEEEARKVKESAEE<br>QGDSEVKRLAEEAEQLAREARRHVQEC<br>RGGWLEH
C6-71-9-1	Oligomer fusion	PEEILERAKESLERAKEAFERGDEEEFR<br>KAAEKALELAKRLVEQAKKEGDPELVFE<br>AARVALWVAWLAAWFGDKEVFKKAAES<br>ALEVAKRLVEVAKEEGDPELVLKAAFVAL<br>LVAIMAVILGDKEVFKKAAESALEVAKRL<br>VEIAAREGDPELVEEAAKVAELVRELAKL<br>MGDEEVYEKARETAREVRLFLLFVRIWE<br>GGG
C6-79-10	Oligomer fusion	SSDEEEARELEERAREAAKRAIEAAKRT<br>GDPRVRELAEELVKLAIWAAVEVWLDPS<br>SSDVNEALKLIVEAIEAAVRALEAAERTG

		DPEVRELARELVRLAVEAAEEVQRNPSS SDVNEALKLIVIAIEAAVRALEAAERTGD PEVRELARELVRLAVEAAEEVQRNPSSE EVNEALRKIIKLILFAVMVLELAEEIGDPT WREMARRAVREAVELAEEVQRDPSGW LGH
C8-71-7-1	Oligomer fusion	PEEILERAKESLERAREASERGDEEEFR KAAEKALELAKRLVEQAKKEGDPELVLE AARVALWVAELAAKNGDKEVFKKAAES ALEVAKRLVEVASKEGDPDLVAWAALVA LWVAFLAFLNGDKEVFKKAAESALEVAK ALMEVAMKVGAPWLVELAIAVARAVWLL AELFGDEEVRRRAEAFEIILRIAAIAVKAW LGGG
H6	Oligomer fusion	TEDEIRKLRKLLEEAEKKLKKLEDKTRRS EEISKTDDDPKAQSLQLIAESLMLIAESLL IIAISLLLSSR
H8	Oligomer fusion	SAEELLRRSREYLKKVKEEQERKAKEF QELLKELSERSEELIRELEEKGAASEAEL ARMKQQHMTAYLEA

The DMX pipeline, on the other hand, is not demonstrated on a particular example. The authors claim that this strategy is more cost-effective for >400 constructs, although it requires a substantial amount of work, for coding and decoding sequences, while the functional characterization of each variant remains to be done in the same manner as for other designs (SEC seems difficult to perform for 2000 variants ?).

We thank the reviewer for the comment. We do present a complete end-to-end demonstration using: "*a library of 1500 de novo designed protein with gene lengths ranging from 275 - 400 bp. We processed 4608 clones, recovering 78% of library variants as clonal, sequence-verified constructs, approaching the theoretical maximum recovery rate of 92% given the library complexity*" (fig. 3c). The Sankey diagram (fig 3c) provides the pipeline's efficiency at each step (barcoding completeness, gene correctness, clonality). We also provide wa detailed cost comparison showing 5-8x cost reduction over individual gene fragments (fig. 3, sup table 5). The output of DMX is arrayed, expression-ready, sequence-verified clonal culture in 96 or 384-well plates, which can be directly processed into the SAPP pipeline for protein production and characterization.

The reviewer expresses concern that SEC may be difficult to perform for 2,000 variants. At <5 min per sample, processing 192 SEC is an overnight run, which is routine at our institute, and in other labs who have already adopted this protocol, This represents >1000 SEC per week on a single AKTA or HPLC.

The sequence-verified plasmids from DMX can be used not only for recombinant protein expression but also for other downstream applications, among them more complex gene construction, transfection, or cell-free synthesis.

In practice, the SAPP/DMX protocols have already been used to characterize tens of thousands of de novo designed proteins at our institute and laboratories across the world, as well as companies, spanning dozens of projects (refs 1,4,5,8,10,40,52-65).

Of note, multiple academic laboratories have already requested DMX vectors and barcodes to incorporate this method into their own research pipelines.

Other strategies, such as e.g. ribosomal or RNA-display, or encapsulation based strategies might be better suited to match each sequence for a protein phenotype in much more effective ways than this protocol. In that type of assay, only the selected variants need to be sequenced.

The reviewer suggests display technologies such as ribosomal- or RNA- display, or encapsulation-based strategies may be better suited for matching sequence to phenotype. We agree that these are powerful methods for their intended applications, and we acknowledge this in the beginning of our manuscript (line 41-46): "Pooled assays can test thousands or even millions of designs in parallel […] providing a compelling alternative to arrayed testing when large amounts of data are required for a project. However, these assays are usually highly specialized towards measuring one or only a few properties (e.g. stability, binding, enzymatic activity), require significant upfront protocol development, and rarely provide information on the manufacturability of individual sequences (expression, solubility, dispersity)."

DMX/SAPP and pooled display methods are complementary, not competing approaches; both have distinct use cases. While indeed, display technologies might be appropriate for certain use-cases, others may require full expression/purification of variants at scale as part of validation campaigns (e.g. for manufacturability, structural, solubility, dispersity or neutralization studies), which DMX/SAPP enables. Our manuscript describes methods for situations specifically requiring purified protein material and arrayed characterization, and we think this difference is clearly shown in the introduction. We have added a brief discussion in the revised manuscript to further clarify the complementary roles of pooled vs arrayed approaches: (p 9) "The workflows both democratize and accelerate protein design,

making large-scale experimental testing more affordable and accessible, and complement existing pooled screens such as display technologies.”
